# Identification and characterization of tumor-associated astrocyte subpopulations and their interactions with the tumor microenvironment in experimental glioblastomas

**DOI:** 10.1371/journal.pbio.3002893

**Published:** 2025-10-13

**Authors:** Mitrajit Ghosh, Paulina Pilanc-Kudlek, Szymon Baluszek, Karol Jacek, Katarzyna Poleszak, Paulina Szadkowska, Anna M. Lenkiewicz, Bartłomiej Gielniewski, Aleksandra Ellert-Miklaszewska, Maria G. Castro, Bozena Kaminska

**Affiliations:** 1 Laboratory of Molecular Neurobiology, Nencki Institute of Experimental Biology of the Polish Academy of Sciences, Warsaw, Poland; 2 Sequencing Core Facility, Nencki Institute of Experimental Biology of the Polish Academy of Sciences, Warsaw, Poland; 3 Department of Neurosurgery, University of Michigan Medical School, Ann Arbor, Michigan, United States of America; 4 Department of Cell and Developmental Biology, University of Michigan Medical School, Ann Arbor, Michigan, United States of America; Luxembourg Institute of Health, LUXEMBOURG

## Abstract

Astrocytes comprise ~50% of all brain cells and present distinct morphological, molecular and functional properties in different brain regions. In glioblastoma (GBM), an aggressive primary brain tumor, astrocytes become activated and tumor-associated astrocytes (TAAs) exhibit different transcriptomic profiles, morphology, and functions supporting disease progression. Heterogeneity and specific roles of TAAs within various regions of tumors are poorly known. Advancements of single-cell and spatial transcriptomics allow to profile tumors at unprecedented resolution revealing cell phenotypes, hidden functionalities, and spatial architecture in disease-specific context. We combined spatial transcriptomics and multiple immunofluorescent staining to visualize TAAs heterogeneity and location of various subpopulations in three intracranial murine glioma models. Using distinct gene expression profiles, we identified subtypes of TAAs with distinct localization and inferred their specialized functionalities. Gene signatures associated with TAAs reflected their reprograming in the tumor microenvironment (TME), revealed their multiple roles and potential contributing factors shaping the local milieu. Using spatial correlation analysis of the spots, we inferred the interactome of *Slc1a2* (encoding a glutamate transporter) with the other markers of TAAs based on segregated areas of the tumor. The designer RGD peptide that blocked tumor-microglia communications, altered the spatial distribution of TAAs in GL261 gliomas providing new insights into cell-to-cell communication. Spatial transcriptomics combined with multiple staining unveiled multiple functional phenotypes of TAAs and interactions within TME. Altogether, we demonstrate distinct morphology of TAAs and different roles in various regions of the tumor. Glioma-induced heterogeneity of TAAs allows adaptation to the pharmacologically induced modification of the immunosuppressive TME.

## Abstract 2

In glioblastoma, astrocytes become activated and tumor-associated astrocytes (TAAs) have different transcriptomic profiles and morphology, as well as unclear functions in supporting disease progression. This study combines spatial transcriptomics with immunofluorescence to identify and characterize distinct TAA subtypes with specific adaptive responses to tumor microenvironment modifications.

## Introduction

Glioblastoma (GBM) is the most deadly, primary brain tumors in adults. The median survival of GBM patients treated with multimodal therapy, including surgery, radiation therapy, and chemotherapy, is only 15 months and did not changed in last 20 years [1]. The tumor microenvironment (TME) of GBM is heterogeneous in cell composition, highly immunosuppressive, and spatially organized. Tumor-associated immune and stromal cells modulate tumor progression, impact therapy outcomes, and survival of patients [2–6].

Astrocytes are most abundant cells in the brain (they comprise ~50% of all brain cells) and perform critical physiological functions maintaining homeostasis of fluids and neurotransmitters. They control calcium signaling, synapse maintenance, and metabolic supply to support active neurons [7,8]. The molecular and cellular heterogeneity of astrocytes is poorly defined, mostly by morphological categories as fibrous and protoplasmic astrocytes expressing GFAP (glial fibrillary acidic protein). Combination of transgenic mice, fluorescence-activated cell sorting followed by bulk RNAseq revealed distinct morphological, molecular, and functional properties of astrocytes from different brain regions [9]. Astrocytes are important part of the blood–brain barrier (BBB), as they physically interact with blood vessels through their endfeet and secrete factors that influence the BBB integrity and function [10–12]. They are gatekeepers from peripheral insults [13–16] and respond to acute insults by undergoing an inflammatory transition to “reactive” state followed by creating a glial scar separating the damaged from the intact brain [17–19]. A high-resolution transcriptomic and spatial cell-type atlas of the whole adult mouse brain was recently created by combining single-cell RNA sequencing (scRNA-seq) from anatomically defined regions and spatial transcriptomics using multiplexed error-robust fluorescence in situ hybridization. It shows the distinct cell-type organization in different major brain structures and considerable heterogeneity as some regions contain cell classes and types highly distinct from those present in the other parts of the brain [20]. It suggests that astrocytes exhibit region-specific transcriptional profiles and distinct pattern of activation depending on a disease.

Tumor-associated astrocytes (TAAs) are the abundant cells among the GBM stromal compartment and actively interact with GBM cells through diverse cross-talks [21,22]. Reactive TAAs play a crucial role in GBM TME [7,19], certain molecularly defined TAA subpopulations were found in mouse gliomas and analogous populations in primary human brain tumors [9]. Two spatially distinct populations of TAAs were identified in mouse gliomas, with those at the tumor periphery being different than those in the perivascular niche [23]. TAAs surrounding the tumor resembled morphologically reactive astrocytes, while CD44 and Tenascin-c-expressing TAAs were restricted to the perivascular niche. Genetic lineage tracing and fate mapping of astrocytes showed that distinct subpopulations expressing GFAP or GLAST (glutamate aspartate transporter) support regional glioma growth in different manner which unravels various functions of TAAs in the spatial context [24]. The brain with its cellular complexity and spatial organization represents a unique microenvironment, and the mechanisms by which the glioma cells interface with resident populations are not fully defined. While the results of scRNA-seq studies expanded our understanding of gliomas by capturing various malignant cellular states and a vast range of nonmalignant cell types in human and experimental gliomas [25,26], subclasses of TAAs and their regional localization in GBM are poorly demarcated.

Spatial transcriptomics technologies allow the acquisition of gene expression information from intact tissue sections in the original physiological context at high resolution. Despite some drawbacks such as not achieving single-cell resolution with spot-based spatial transcriptomics or not detecting efficiently some states that are spatially scattered or lowly abundant by unsupervised analysis [27], these techniques allow better resolution of the architecture of normal brain and tumor.

In this study to dissect the transcriptional diversity of TAAs in a spatial context in three mouse glioma models, we combined spatial transcriptomics and immunohistochemistry (IHC) which validated candidate proteins with high cell type specificity. We demonstrate discrete subpopulations of TAAs exhibiting various functional states of tumor-driven activation in spatially resolved locations, and provide insights into the relevance of glial scar in malignant gliomas. Moreover, we show that the designer RGD peptide that block reprograming of myeloid cells in the TME and normalizes a vascular niche, affects distribution of TAAs. Altogether, the data provide valuable insights on spatial aspects of transcriptomic heterogeneity of astrocytes in experimental gliomas and show consequences of the therapeutic modulation of tumor–host interactions that subsequently changes the tumor niche.

## Materials and methods

### Animals

Male C57BL/6J 10–12 weeks mice (Charles River Laboratories, USA) were used for experiments. Mice were housed with free access to food and water, on a 12 h/12 h day and night cycle. All efforts have been made to minimize the number of animals and animals suffering. All research protocols conformed to the Guidelines for the Care and Use of Laboratory Animals (European and national regulations 2010/63/UE September 22, 2010 and Dz. Urz. UE L276/20.10.2010, respectively). Animals were decapitated by a qualified researcher. The First Warsaw Local Ethics Committee for Animal Experimentation approved the study (approval no. 812/2019;1049/2020).

### Glioma cell cultures

GL261 glioma cells were obtained from prof. Helmut Kettenman (MDC, Berlin, Germany) and GL261 tdT+luc+ glioma cells which stably express Firefly Luciferase (luc) and tandem Tomato (tdT) fusion fluorescent protein were generated as previously described [28]. GL261 tdT^+^luc^+^ cells were cultured in Dulbecco’s modified Eagle’s medium (DMEM) supplemented with antibiotics (100 U/ml penicillin,100 µg/ml streptomycin) and 10% fetal bovine serum (FBS) (Gibco, MD, USA), Cells were cultured in a humidified atmosphere of CO_2_/air (5%/95%) at 37 °C (Heraeus, Hanau, Germany). Two glioma cell lines with specific genotype: (1) NRas; shTP53-GFP; shATRX; wtIDH1 and (2) PDGFB; shTP53; shATRX; Ink4a; Arf^−/−^; wtIDH1 were generated by prof. Maria G. Castro. Those cells were cultured as floating spheres in DMEM/F12 (Gibco, 31331-028), B-27 supplement (1×; Gibco, 12587-010), N-2 supplement (1×; Gibco, 17502-048), Antibiotic Antimycotic (100×) Streptomycin Amphotericin B Penicillin (Gibco; 15240062), Normocin (1×; InvivoGen, ant-nr-1), hFGF and hEGF (20 ng/μL each stock, 1,000× formulation; Shenandoah Biotech, 100-26, 100-146) in a humidified atmosphere of CO_2_/air (5%/95%) at 37 °C (Heraeus, Hanau, Germany).

### Stereotactic implantation of glioma cells and treatments

Ten-week-old male mice (C57BL/6J) were anesthetized with isoflurane (4%–5% induction, 1%–2% maintenance) using Isoflurane vaporizer (Temsega, Tabletop Anesthesia Station). Before starting the surgical procedure and during the surgery, the depth of anesthesia was verified by the lack of deep pain response in the limb. Choice of specific anesthetics was recommended by the veterinarian and approved by The Local Ethics Committee. After identifying the sagittal and coronal sutures on the right side, a hole was drilled at the following coordinates: 1 mm anterior and 2 mm lateral from bregma. Tumor cells (80,000 in 1 μL of DMEM) were stereotactically injected with a Hamilton syringe to the right striatum 3 mm deep from the surface of the brain.

For the experiments with RGD/control peptides, Alzet osmotic micropumps (DURECT Corporation, Cupertino, CA, USA) were installed at the time of injection in a subcutaneous pocket on the back, slightly caudal to the scapulae. A small incision was made in the shaved skin, and a hemostat was used to create a subcutaneous pocket for the pump, which was then inserted into the pocket, and the wound was closed with tissue glue. The skin was closed and mice were monitored until they completely recovered from anesthesia. To ensure that the pumps were active when implanted, the filled pumps were placed in sterile saline at 37 °C for 24 h before implantation. Osmotic pumps were filled with H_2_O (vehicle), 7aaRGD or 7aaRAE peptides at a concentration of 2 mg/ml in H_2_O, and by means of a catheter, they continuously delivered the solutions intratumorally at a controlled rate of 0.11 µL/h for 21 or 28 days.

In all experiments, the animals were weighed weekly and observed daily for clinical symptoms. Tumor growth was verified by assaying the luciferase activity of implanted glioma cells using Xtreme in vivo bioluminescence imaging system (Bruker, Germany) at 7, 14, and 21 days post-implantation.

### Visium spatial transcriptomics

#### Sample preparation and tissue optimization.

Mice were anesthetized and sacrificed by transcardial perfusion with phosphate-buffered saline (PBS). Brains from naïve or tumor-bearing mice were removed and snap-frozen in tissue freezing medium (Leica, Ref. 14020108926) on dry ice. Brains were coronally sectioned to 10 μm using a cryostat (Thermo Scientific, Microm HM525) at −20 °C and mounted onto the etched fiducial frames of the Visium Tissue Optimization Slide according to the Tissue Preparation Guide (CG000240, Rev E, 10× Genomics). A single brain hemisphere section per mouse was mounted on Visium Spatial Gene Expression Slides (catalogue no. 2000233, 10× Genomics). Sections were fixed with pre-chilled methanol at −20 °C for 30 min. Hematoxylin and eosin (H&E) staining was performed and subjected to bright-field imaging under a Leica DM4000B microscope according to the staining protocol and imaging guidelines (CG1000160, CG000241, 10× Genomics). Raw images were acquired with a Leica DM4000B microscope and exported as tiff files. Sections were permeabilized with the Permeabilization Enzyme for different times. The released mRNA was captured by probes on the slides, and reverse transcribed to cDNA marked by fluorescently labeled nucleotides. Tissue was then removed from the slides with a digestive enzyme, leaving the fluorescently labeled cDNA, which was visualized under a Leica DM4000B microscope according to Tissue Optimization Guide (CG000238 Rev E, 10× Genomics). Based on the signal intensity, we determined that the optimal permeabilization time for a tumor-bearing mouse brain is 26 min. Total RNA was extracted from frozen brains tumor using the RNeasy Kits according to the manufacturer’s instructions (Qiagen). Size, quantity, integrity, and purity of all samples were measured using the 2100 Bioanalyzer instrument and RNA chip (Agilent). All sections had an RNA integrity number >8. RNA was eluted in 50 µl RNase-free water and stored at −80 °C until transcriptome profiling.

Visium spatial gene expression library construction and sequencing: Visium spatial gene expression slides and reagents kits (10× Genomics; #1000187, Dual Index Kit TT Set A; #1000215) and RNeasy Kit (Qiagen; #74134) were used according to manufacturer instructions (10× Genomics). Sections were fixed in methanol at −20 °C for 30 min and stained for H&E (Sigma-Aldrich; #SLCJ5200, #SLCH5595) for general morphological analyses and spatial alignment of sequencing data. After bright-field imaging, brain sections were enzymatically permeabilized for 26 min, stained and fixed tissue sections were deposited onto the slide. The poly-A mRNA was released from slide covering cells and was captured on each of the spots on the capture area. Each slide spot contained the special barcode composed of an Illumina compatible Truseq sequence, unique barcode, UMI, and poly(dT) sequence. Library preparation was done according to the Visium Spatial Gene Expression User Guide (CG000239, Rev G, 10× Genomics). The concentration of the resulting libraries was determined by a Quantus Fluorometer with a QuantiFluor ONE Double-Stranded DNA System (Promega, Madison, WI, USA), and the quality check was performed using an Agilent Bioanalyzer (Agilent Technologies, Santa Clara, CA, USA). The obtained libraries were pooled together and mixed to achieve sequencing depth recommended by manufacturer instructions based upon the slide coverage. Sequencing was performed on Novaseq 6000 (Illumina, San Diego, CA, USA) with the recommended protocol (read 1: 28 cycles; i7 index read: 10 cycles; i5 index read: 10 cycles; and read 2: 50 cycles), yielding between 150 and 224 million sequenced reads. The eight dual-index Illumina paired-end libraries were sequenced on a NovaSeq 6000 on an S2 100-cycle flow cell using 150 base pair paired-end dual-indexed set-up to obtain a sequencing depth of ~50,000 reads as per 10× Genomics recommendations. BCL to FASTQ conversion was performed using SpaceRanger (v1.2.0). Raw FASTQ files alignment to the 10× Genomics mouse reference genome GRCm39–2020 and the reads assignment to spots was performed using SpaceRanger (v1.2.0). The expression matrix was normalized and scaled using the NormalizeData and ScaleData functions from Seurat package (v4.3.0) [29]. Next, squared coefficient variance (cv2) modeling was utilized to select the most variable genes, principal component analysis (PCA) was run, and the Marchenko–Pastur algorithm was utilized to identify nonrandom [30] components. These data were integrated with canonical component analysis in Seurat IntegrateData function. Subsequently, again PCA was run, Marchenko–Pastur algorithm was applied, to uniform manifold approximation and projection (UMAP) was derived, and clustering with the Leiden algorithm was performed [30].

#### Spatial gene expression, profiles, and correlation analysis.

Differential gene expression between clusters was performed in pairs: tumor core–tumor periphery, tumor periphery–border area, and border area–normal brain. Effect size (log2(fold-change)) calculated between areas using wilcoxauc function from presto package (v1.0.0) was an input for a gene-set enrichment analysis (GSEA) on gene sets from Gene Ontology [31] and Reactome [32] databases. The GSEA was performed using the fgsea algorithm from fgsea package (v.1.24.0).

Gene pairs correlation was computed using the Spearman test within each group of spots (brain, peri-tumoral area, tumor border, and core); *p*-value adjustment was performed with the Benjamini–Hochberg procedure. For comparison of correlation coefficients in each area, Fisher transformation was applied to correlation coefficients and *t*-distribution was utilized for hypothesis testing (H0: the correlation coefficients are not different, H1: the correlation coefficients are different, *α* = 0.05).

To evaluate the stability and statistical significance of the pairwise correlations between the signals, we employed a bootstrap procedure: **Resampling:** For each of a specified number of replicates (default: 1,000), the dataset was resampled with replacement. **Correlation Computation:** In each replicate, a Pearson correlation matrix was computed using the resampled data. **Aggregation:** Across all replicates, the mean correlation coefficient for each cell type pair was calculated. Additionally, the lower and upper bounds of the confidence intervals were derived using the quantiles corresponding to the chosen significance level (*α* = 0.05). **Significance Indicator:** A binary indicator was assigned to each cell type pair, flagging those for which the confidence interval did not cross zero—indicating a statistically robust co-occurrence of signals.

This algorithm was applied separately to data extracted from different tumor regions (tumor core, tumor periphery, and border), enabling a detailed investigation of spatially variable interactions among the cellular signals.

For method validation, we employed the publicly available 10X Visium platform samples from GSE194329 [33]. Data alignment from SpaceRanger was available and utilized. Spatially aware data integration was applied from Banksy [34]. Clustering was conducted using Seurat 29,30] with Leiden clustering (resolution 0.75) applied to identify distinct transcriptional regions. Comparative analysis with murine data involved scaled cosine similarity metrics to assess cross-species conservation of TME features. Dot plot was utilized for the visualization of features of interest.

### Immunofluorescence

The animals were sacrificed 21 days after tumor cell implantation and perfused with 4% paraformaldehyde in PBS. Brains were removed, post-fixed for 48 h in the same fixative solution, and placed in 30% sucrose in PBS at 4 °C until they sunk to the bottom of the flask. Tissue was frozen in Tissue Freezing Medium (Leica Biosystems, Richmond, IL, USA) and cut in 10 µm coronal sections using a cryostat. The slides were thawed and dried for 5 min at 37 °C after being transferred from the −80 °C storage. Next, the slides were put in pre-chilled methanol at −20 °C for 30 min. The cryo-sections were washed 3× for 5 min in PBST (0.1% Triton X-100 in PBS), blocked with 3% DS in 0.4% Triton X-100 in PBS for 2 h at RT, stained overnight with primary antibodies at 4 °C followed by 3× washes in PBST and incubation with secondary antibody for 2 h at RT. All antibodies were diluted in 0.4% Triton X-100/PBS solution containing 3% donkey serum. Sections were washed 3x in PBST for 5 min, once in PBS for 5 min, followed by washing in ultrapure water for 5 min and mounted with Vectashield Vibrance Antifade Mounting Medium (# ZKO8O3, Vector Labs, US) with or without DAPI (0.001 mg/ml), for counterstaining nuclei. Images were obtained using the Leica DM4000B fluorescent microscope. Following primary antibodies were used for IF staining: anti-GFAP (DAKO; #Z0334), anti-ALDH1L1 (Abcam; #ab177463), anti-S100B (Abcam; #ab52642), anti-Glutamate Transporter (Chemicon; #ab1783), anti-NeuN (Chemicon; #MAP377), anti-TMEM119 (Synaptic Systems; #400004), anti-fibronectin (Merck; #ab2033), anti-laminin (Abcam; #ab11575), anti-transglutaminase2 (Thermo scientific; MA5-12915). Secondary antibodies such as donkey anti-rabbit IgG Alexa Fluor555 (Invitrogen; A31572), donkey anti-rabbit IgG Alexa Fluor 488 (Invitrogen; A21206), donkey anti-mouse IgG Alexa Fluor 555 (Invitrogen; A31570), donkey anti-rabbit IgG Alexa Fluor 488 (Invitrogen; A21202), and goat anti-guinea pig IgG Alexa Fluor 488 (Invitrogen; A11073) were used. Primary antibodies were used in 1:50 or 1:100 dilution when secondary antibodies were used in 1:500 dilution. For each staining, three different sections from each mouse brain were analyzed. In each section, 4 ROIs (Region of Interest) were analyzed in and around the tumor core. We quantified the percentage of Gfap+ cells from the DAPI+ cells to get an estimate of area coverage by thresholding in ImageJ.

### Astrocyte cell primary cultures and co-cultures with glioma cells

Primary glial cell cultures were prepared from the cerebral cortices of P0-P2 pups of C57BL/6J mice as previously [6] described. The cells were suspended in the medium, counted, checked for viability, and seeded at a required density in high-glucose DMEM supplemented with Glutamax, 10% fetal bovine serum (ThermoScientific; CA, USA) and antibiotics. Microglia were identified by immunofluorescent staining as Iba1+ cells (>99%), and negative for the astrocyte marker Gfap. The remaining glial cell monolayer was subjected to strong shaking at 180 rpm overnight to remove sparse oligodendrocytes and *>*97% pure astrocyte cultures were [33] obtained. Primary astrocyte cultures were identified by immunofluorescent staining as >99% Gfap+ and negative for the microglia marker Iba1.

Astrocytes (2.5 × 10^6^ cells) were seeded in polylysine (PLL)-coated 6-well plates in 2 ml of the DMEM Glutamax medium. Astrocyte cultures were co-cultured with glioma cells 48 h after seeding. Glioma cells were seeded on inserts with 0.4-mm pores at density 2.5 × 10^6^/insert. After 24 h, the medium was changed to an astrocytic medium, and these inserts were transferred into the plate with astrocytes for another 24 h. The glass coverslips were used for immunofluorescence (IF) or cell lysates were collected for protein analysis by Western Blotting.

### Western blotting and quantification

Whole-cell protein extracts were prepared, resolved by electrophoresis, and transferred to a nitrocellulose membrane (GE Healthcare, #10600003). After blocking with 5% nonfat milk in TBST (Tris-buffered solution pH 7.6, 0.01% Tween-20), the membranes were incubated overnight with primary antibody anti-Tgm2 (Invitrogen, #PA5-29356, 1:500) or anti-Laminin (Abcam, #11575 dilution 1:2,000) diluted in a TBST with 5% nonfat milk or with anti-GAPDH (Merck, #MAB374) diluted 1:25,000 in 5% bovine serum albumin (BSA) in TBST. The primary antibody reaction was followed by 1 h incubation with horseradish peroxidase-conjugated anti-rabbit IgG (Vector, #PI-1000) or anti-mouse (Vector, #PI-2000) which were diluted 1:10,000. Immunocomplexes were detected using an enhanced chemiluminescence detection system (ECL) and Chemidoc (Biorad). Molecular weight of proteins was estimated with the Cozy pre-stained protein ladder (High Qu GmbH, #PRL0102c1). Band intensities were measured by densitometry of immunoblots with BioRad Image Lab software.

### Statistical analysis

Each experiment was performed at least three times, on independent passages/cultures, at least in duplicates. For spatial transcriptomics study, *n* = 2 mice per model and condition was used, and for IF studies, *n* = 4–5 for each group was used. Statistical analyses were performed using GraphPad Prism v6.01 (GraphPad Software, San Diego, CA, USA). The data were plotted as mean ± SD. Differences between the means of the treatments were evaluated using one-way analysis of variance (one-way ANOVA) followed by post hoc Dunnett’s multiple comparison test or one-sided paired sample *t* test (*p*-value) for two groups analysis. *P*-values were calculated using GraphPad software and considered significant when *P < 0.05 (one-way paired *t* test).

## Results

### Tumor-associated astrocytes exhibit spatially organized gene expression in experimental gliomas

**Fig 1:**
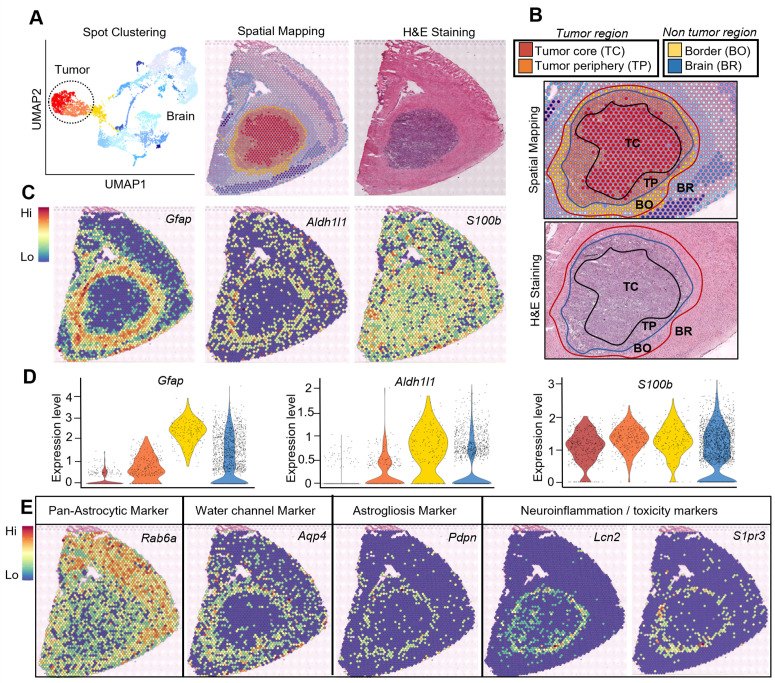
Dissecting the transcriptional heterogeneity of tumor-associated astrocytes in experimental mouse gliomas. **(A)** UMAP showing brain and tumor cell projection by spot clustering, spatial mapping on the tissue section with the Visium 10× Genomics platform and matching with H&E staining from the same tissue section. **(B)** Identification and segregation of distinct areas being readouts from UMAP on spatial projection as the tumor core (TC), tumor periphery (TP), border (BO), and brain regions (BR). **(C)** Spatial mapping of astrocyte marker genes (*Gfap*, *Aldh1l1*, and *S100b*) on tissue sections from Visium gene expression readout. **(D)** Violin plots showing expression profiles of *Gfap*, *Aldh1l1*, and *S100b* at different tumor and nontumor regions of the tumor microenvironment. *n* = 2 per condition. **(E)** Spatial mapping of *Rab6a* (pan astrocytic marker), *Aqp4* (water channel marker), *Pdpn* (astrogliosis marker), *Lcn2* and *Sipr3* (neuroinflammatory markers).

To investigate the TAAs landscape and explore their distinct spatial location within glioma, we used the 10× Visium spatial transcriptomics platform and IF to validate the results at a single cell level (Fig 1A). In the experiment, 12 fresh frozen mouse brains were analyzed, of which 4 samples (2 control and 2 tumor–bearing brains) were used for the Visium and 8 samples (4 control and 4 tumor–bearing brains) for the IF studies. Using spot clustering and spatial mapping in GL261 gliomas, we identified the tumor core from the UMAP and mapped on the tissue section stained with H&E (Fig 1A). To better visualize expression pattern of astrocytic markers in the tumor relative to the surrounding tissue, we identified histologically distinct areas such as the tumor core (TC), tumor periphery (TP), border (BO), and nontumor brain (BR) on the tissue sections (Fig 1B).

We located spots with high expression of well-known astrocyte-specific genes such as *Gfap*, *Aldh1l1* (coding for Aldehyde dehydrogenase 1 family L1, robustly expressed on both body and processes of astrocytes) and *S100b* (coding for S100 calcium binding protein B, abundantly expressed in astrocytes) [16,8,35] and mapped them on the tissue section in distinct areas of the brain section (Fig 1C). Cells expressing *Gfap* and *Aldh1l1* localize at the tumor border and form a ring around tumor core consistent with histologically defined “glial scar”, while the expression of *S100b* is uniformly dispersed in the whole brain section. Using the defined area segregation, we demonstrate distinct expression profiles of *Gfap*, *Aldh1l1* and *S100b* in selected areas shown as violin plots (Fig 1D). This spatial mapping was extended to other marker genes and genes associated with functions of astrocytes. *Rab6a* (coding for Ras-related protein Rab6a, a pan astrocytic marker) [36] was abundantly expressed, particularly in the cortex, with some expression in the tumor core. In contrast, *Aqp4* (coding for Aquaporin 4, an important water channel marker) was richly expressed in astrocytes [8,16,34]. *Pdpn* (coding for Podoplanin that has been linked with astrogliosis) [37,38], *Lcn2 (*coding for Lipocalin 2) [39], and *Sipr3* (coding for Sphingosine phosphate receptor 3) which are associated with the immunosuppressive activity of astrocytes [40], formed the ring around the tumor similar to *Gfap* (Fig 1E). Several other important marker genes associated with diverse functions of astrocytes such as *Drd1*, *Spi1*, *Timp1*, *Igtp*, *Cxcl10* clearly demonstrate the spatial heterogeneity in glioma-bearing brains; many were not expressed in the brain of naïve mice (S1 Fig). We also performed spatial transcriptomics analysis of distinct astrocyte markers and expression profiles in NRas and PDGFB mouse gliomas. The results show that *Gfap*, *Aldh1l1*, and *S100b* in NRas gliomas depict similar expression pattern as seen in GL261 gliomas. *Gfap* was expressed in glial scar around the tumor, particularly in NRas gliomas, while expression of *Aldh1l1* and *S100b* was more widely distributed, except for the tumor core (S2 Fig). *Tgm2* (coding for transglutaminase 2) was overexpressed in the tumor core, more strongly in NRas gliomas than in PDGFB mouse gliomas (S2 Fig).

### Morphology of TAAs varies in different tumor areas

**Fig 2:**
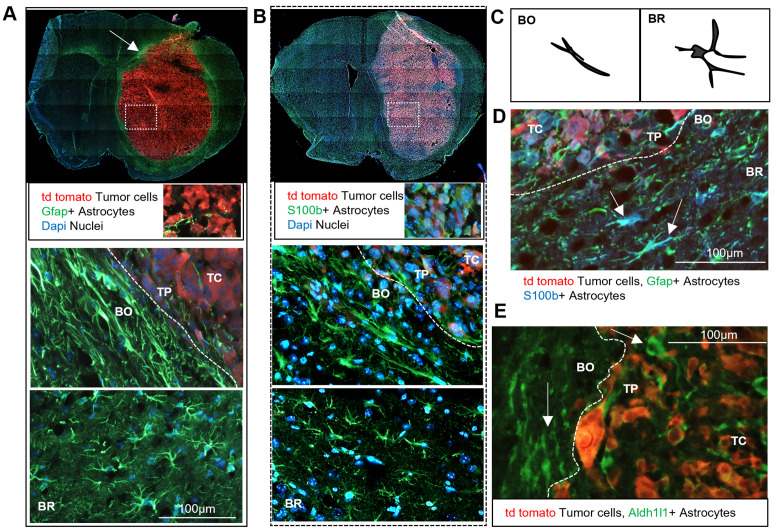
Distinct morphology of astrocytes in various tumor areas. **(A)** Immunofluorescence (IF) staining of mice brain sections showing td tomato labeled tumor cells (in red), Gfap+ astrocytes (in green) and DAPI stained cell nuclei (in blue) depicts a “glial scar’” composed of reactive astrocytes surrounding the tumor core. Gfap+ astrocytes are barely present in the TC (in the magnified inset), show elongated shapes at the BO and star-like morphology in the surrounding brain parenchyma, BR. **(B)** IF staining of S100b+ astrocytes (green) shows different patterns, with a uniform, widespread distribution in the brain and the presence of S100b+ astrocytes in the TC and at the BO. S100b+ astrocytes in the BO are elongated, while S100b+ astrocytes farther from the TC display star-like morphology. **(C)** The scheme illustrates a distinct morphology of astrocytes in BO and BR areas. **(D)** Double IF staining shows S100b+ and Gfap+ bipolar-extended astrocytes at the BO, whereas in the BR astrocytes display radial-star shapes. **(E)** IF staining shows Aldh1l1+, bipolar-extended astrocytes (in green) in the TC and BO regions (indicated by arrows); *n* = 4 per condition.

Activation of astrocytes is associated with distinct morphological changes such as hypertrophy, elongation and process extension [8]. Most of an astrocyte surface area is formed by branches, branchlets and leaflets, and a fraction of it can be estimated by Gfap immunostaining [8,16]. We performed IF staining for several astrocyte markers on sections of glioma-bearing brains. The pattern of Gfap staining was similar as on the spatial map, with Gfap+ cells surrounding the tumor core and forming a glial scar with the highest expression in the border (BO) area (Fig 2A, upper panel) where the bipolar shaped astrocytes form a thick barrier, with rare cells in the tumor periphery (TP) and almost no Gfap staining at the tumor core (TC) (Fig 2A, lower panel). In the nontumor parenchyma (BR) Gfap+ cells exhibit the star-like morphology of typical astrocytes. Staining for S100b revealed a similar pattern of morphological differences in all the segmented areas (TC, TP, BO, and BR) (Fig 2B). Some S100b+ cells were detected within the TC and the ring in BO was not visible, as S100b+ cells showed more homogeneous distribution. Interestingly, in both cases TAAs close to the BO showed a bipolar morphology aligned along the tumor border, while the cells farther from the BO had mostly star-like morphology (Fig 2C). Double staining for Gfap and S100b verified the presence of TAAs with double positive staining within and around BO (Fig 2D). Aldh1l1+ cells were detected in the BO area surrounding the tumor and rare positive cells within the TC (Fig 2E). Altogether, immunostaining for astrocytic markers confirmed distinct distribution of TAAs characterized by expression of different markers within glioma-bearing brains and corroborated data acquired with the spatial transcriptomics.

### Neuron depletion, glutamate dysregulation, and reduced astrocyte signatures in the tumor core

**Fig 3:**
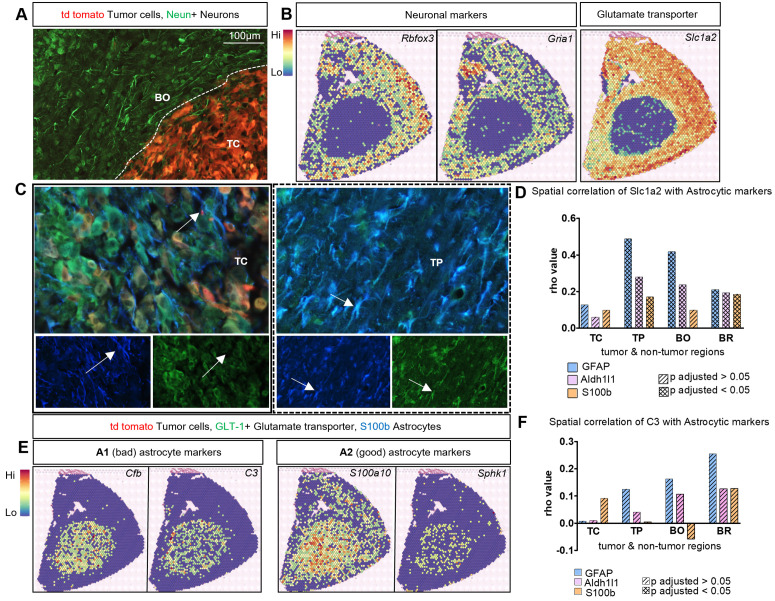
Loss of neurons and glutamate dysregulation coincides with A1 and A2 astrocyte signatures at the tumor core. **(A)** NeuN+ neurons (in green) are absent in the tumor core demarcated by the presence of td tomato tumor cells (in red), while NeuN+ neurons are distributed uniformly outside of the TC. **(B)** Spatial mapping of neuronal markers such as *Rbfox3, NeuN*, *Gria1*, and glutamate transporter 1 marker *Slcia2/Glt1* in tissue sections confirms a lack of neuronal gene expression and *Slcia2* at the TC. **(C)** IF staining of GLT-1 (in green) and astrocyte marker S100B (blue) in mouse brain section with td tomato tumor cells (in red). The arrows indicate numerous double-positive GLT-1+ and S100B+ astrocytes at the tumor periphery (TP) whereas no double-positive cells are visible within the TC; separate channels representing the indicated co-staining. This shows dysregulation of GLT-1 at the TC but not at TP; *n* = 4 per condition. **(D)** Spatial correlation extracted from the Visium data indicates stronger correlation between astrocyte genes such as *Gfap*, *Aldh1l1*, and *S100B* with *Slc1a2* at the TP, BO, and BR compared to the TC; *n* = 2 per condition. **(E)** Spatial mapping of A1 astrocyte-specific genes (*Cfb, C3)* and A2 astrocyte-specific genes (*S100a10, Sphk1*) on tissue sections from the Visium gene expression readout. **(F)** Spatial correlation from the Visium gene expression data indicates correlation of cells expressing *C3* with astrocytic markers at different tumor regions. The underlying data is available at S1 Data.

In response to pathological conditions astrocytes undergo morphological, molecular, and functional changes. Although, there is no a prototypical “reactive” astrocyte, divergence binary phenotypes such as good–bad, neurotoxic–neuroprotective, or A1–A2 is a useful oversimplification. Reactive astrocytes loss some homeostatic functions and gain some protective or detrimental functions, depending on a context, with only a fraction of common changes between different states. To resolve TAAs functionalities, we sought to integrate the information from multiple staining and spatial data. Staining for NeuN (a neuronal marker) showed a complete lack of NeuN+ cells in the tumor core (Fig 3A) consistent with loss of neurons. Cells expressing neuronal marker genes such as *Rbfox3 (*encoding a marker of post-mitotic neurons RNA-binding FOX3 protein)*, Gria1* (encoding glutamate ionotropic receptor AMPA subunit 1) and *Slc1a2* (encoding solute carrier family 1 member 2 also known as Excitatory amino acid transporter 2 - EAAT2) were absent in the TC and TP, while abundant in the BO and the adjacent BR areas (Fig 3B).

There are two main mechanisms of neuronal toxicity and death: (1) glutamate dysregulation leading to enhanced excitotoxicity, and (2) neurotoxicity induced by A1 astrocytes emerging under the influence of microglia [14,19]. The Glutamate transporter 1 (GLT-1) expressed by astrocytes maintains glutamate homeostasis by removing its excess from the extrasynaptic space [41]. Tumor cells produce an excess of glutamate, however, functional astrocytes can maintain its level to support neurons. Cells expressing *Slc1a2* (encoding GLT-1) are less abundant in the TC and TP compared to BO and BR areas on the spatial map (Fig 3B). Double staining for GLT-1 and S100b showed no co-localization of double-positive cells in the TC, while such co-localization was observed in the BO area (Fig 3C). This indicates a potential glutamate dysregulation in the TC as TAAs with lower GLT-1 levels do not efficiently remove excess glutamate which may result in neuronal death.

Using spatial correlation analysis of the spots, we inferred the interactome of *Slc1a2* with the other markers of TAAs based on segregated areas of the tumor. The graph shows interaction profile of TAAs expressing *Slc1a2* and hints that subtypes of TAAs defined by these marker genes may have different activity profiles based on spatial location in the tumor (Fig 3D).

Under pathological conditions, reactive astrocytes acquire distinct phenotypes along the spectrum between the neurotoxic, pro-inflammatory phenotype (A1) and the neuroprotective, anti-inflammatory phenotype (A2) [8,35]. A1 astrocytes are induced by insult-activated microglia that secrete interleukin 1α (Il-1α), tumor necrosis factor (TNF), and complement component 1, subcomponent q (C1q). Their ability to maintain homeostasis and support neuronal survival decreases [14]. Therefore, we investigated the spatial expression of A1 and A2 signature genes such as *Cfb, C3* [A1 markers], and *S100a10, Sphk1* [A2 markers] [14]. Interestingly, all the markers showed clear localization at the tumor site encompassing TC, TP, and BO areas and show low expression at nontumor BR areas; *S100a10* is also expressed in brain parenchyma (Fig 3E). As *C3* represents one of the highly expressed markers for A1 astrocytes, we determined its spatial correlation interactome in the tumor regions where TAAs express *C3* mRNA (Fig 3F), suggesting their neurotoxic capability. We further show correlation of signals from most common cellular entities such as astrocytes and microglia in relation to tumor cells and in the context to different spatial location at the tumor core, periphery, or border (S3 Fig). The underlying data is available at S1 Data.

**Fig 4:**
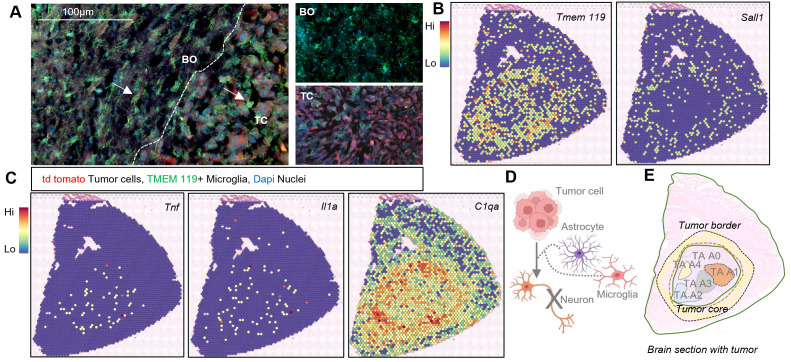
Proximity of microglia influences TAAs in GBM. **(A)** IF staining reveals TMEM 119+ microglia (in green) with a radial shape (surveying microglia) exclusively in BO and BR regions, whereas microglia with ameboid shapes (activated microglia) are present at the TC and mostly within TP regions, close to td tomato tumor cells (in red). In the insets; TMEM119+ microglia in BO and TC regions show morphological diversity; *n* = 4 per condition. **(B)** Spatial mapping of *TMEM119-*expressing microglia in and around the TC, whereas microglia expressing *Sall1* (which is exclusively expressed in surveying microglia) are distributed outside the TC; *n* = 2 per condition. **(C)** Spatial mapping of *Tnfα, Il1β*, and *C1qa* depicts localization in the TC. **(D)** Scheme showing how tumor cells and TAAs influenced by microglia can orchestrate the elimination of neurons at the TC. **(E)** Schematic representation of 5 subtypes of TAAs localized within the tumor-bearing brain based on marker gene expression depicted by spatial transcriptomics and to some extent validated by immunofluorescence.

Microglia, brain resident myeloid cells, induce neurotoxic reactive astrocytes and microglia-derived cytokines are the main inducers of the A1 phenotype [14]. Thus, we determined spatial location of microglia and subpopulations of TAAs in gliomas with ST and IF staining. Tmem119+ nonactivated microglia (with radial processes) were found exclusively in the BO and BR regions, whereas the activated microglia (ameboid shaped) were localized mostly at the TP (Fig 4A). Accordingly, *Tmem119* expression was mapped by ST in a proximity to the tumor core and border. Another microglial marker *Sall1*, which is expressed mostly in the nonactivated microglia [42], shows the uniform expression in the brain parenchyma and no expression at the TC (Fig 4B). Genes coding for inflammatory cytokines such as *Tnf, Il1a* are sparsely distributed in the tumor core*,* while *C1qa* is highly expressed in this region (Fig 4C). Therefore, spatial overlap of microglia and cells expressing cytokines, especially at the TC, suggests that these genes are mainly expressed by microglia and influence TAAs (Fig 4D and 4E), as previous studies with co-cultures demonstrated [14,28].

Altogether, distribution of marker gene expression, functional correlation and localized occupancy suggest the existence on at least five subtypes of astrocytes in GBM: the A0 subtype (expressing *S100b, Rab6a*) which is homogeneously distributed in the brain, the A1 neurotoxic TAAs expressing *Cfb, C3* and overlapping spatially with cells producing pro-inflammatory cytokines; A2 TAAs expressing *S100a10, Sphk1* markers and located mainly at the TC; A3 *Aldh1l1* expressing TAAs localized in the TC and BO, which has utmost importance for functions beyond Gfap reactivity; A4 the “reactive”, *Gfap*, *Aqp4, Pdpn, Lcn2* expressing TAAs that form the scar or barrier with high expression at the BO and minimal expression at the TC (Fig 4E).

### Spatial transcriptomic analysis of human glioblastoma recapitulates the TAAs heterogeneity

**Fig 5:**
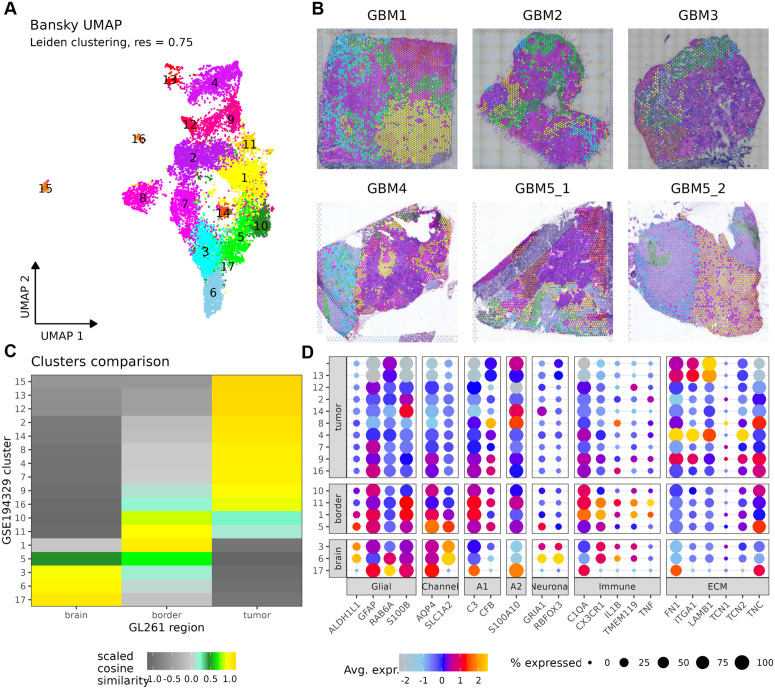
Spatial transcriptomic analysis of human glioblastoma samples confirms distinct tumor microenvironment regions. This figure presents a comprehensive spatial transcriptomic analysis of human glioblastoma samples using the Banksy computational framework. (**A)** UMAP visualization of integrated transcriptomic data from six (*n* = 6) GBM samples, colored by Leiden clustering at resolution 0.75. Distinct clusters are numbered 1–17. **(B)** Spatial distribution of identified clusters across six GBM samples (GBM1-GBM5_2), showing the topographical organization of transcriptionally distinct regions within each tumor section. **(C)** Heatmap comparing GBM clusters with mouse GL261 glioma regions, showing the relative similarity (scaled cosine similarity) of human GBM clusters to predefined mouse tumor regions classified as “brain,” “border,” or “tumor,” Clusters 3, 6, and 17 show the highest similarity to normal brain tissue; clusters 1, 5, 10, and 11 correspond to tumor–brain border regions; other clusters primarily match the tumor core *n* = 6. **(D)** Dot plot showing expression of marker genes across identified clusters, organized by functional groups and by spatial region. Dot size represents percentage of cells expressing each gene, while color intensity indicates average expression level. Notable patterns include A1 reactive astrocyte markers in border regions and tumor-specific expression of ECM-related genes, *n* = 6.

Our spatial transcriptomic analysis of human GBM samples explored the public data from GSE194329 [30] using the Banksy computational framework and revealed the distinct TME regions, corroborating findings from the murine GL261 gliomas. UMAP visualization of integrated transcriptomic data from six GBM samples (GBM1-GBM5_2) identified 17 transcriptionally distinct clusters (Fig 5A). Spatial mapping confirmed the topographical organization of these clusters, with clusters 3, 6, and 17 exhibiting high similarity to normal brain tissue, while clusters 1, 5, 10, and 11 corresponded to tumor-brain border regions (Fig 5B and 5C). Dot plot analysis highlighted functional marker gene expression patterns, including A1 reactive astrocyte markers in border regions and tumor-specific extracellular matrix (ECM)-related genes. The “reactive”, *GFAP*, *AQP4, PDPN, LCN2, C3* expressing TAAs are abundant at the tumor border (Fig 5D). These findings align with the results of murine glioma analysis, where *Gfap*, *Aqp4, Pdpn, Lcn2* TAAs form a glial scar around the tumor core, exhibiting region-specific morphologies and gene expression profiles (Fig 5D).

### TAAs contributes to extracellular matrix remodeling

**Fig 6:**
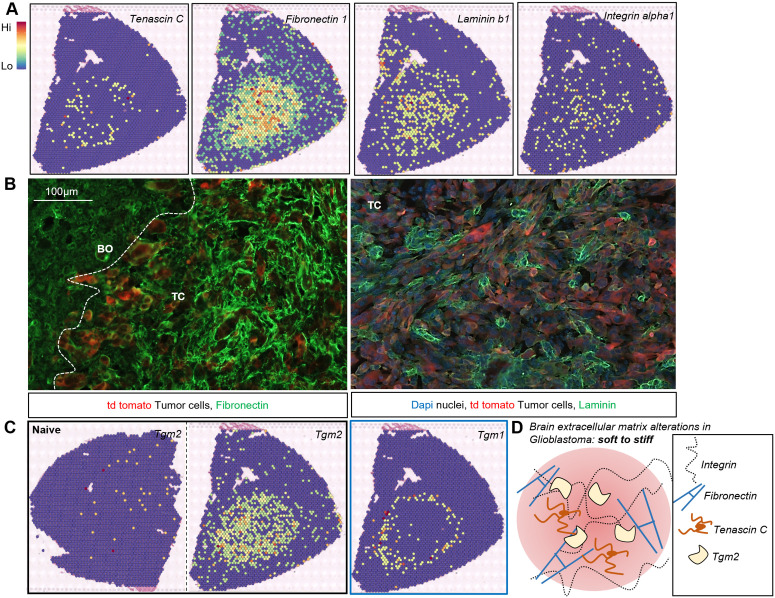
Mapping extracellular matrix remodeling in experimental gliomas. **(A)** Spatial mapping of *Tenascin C*, *Fibronectin 1*, *Laminin b1*, *Integrin alpha1* encoding important extracellular matrix proteins in sections of brains bearing gliomas denotes localization of these markers in and around TC and TP regions. **(B)** IF staining shows upregulation of Fibronectin and Laminin in the TC, in agreement with spatial transcriptomics data. **(C)** Upregulation of *Tgm2* encoding a tissue transglutaminase 2 in sections of brains bearing gliomas whereas there is no expression of *Tgm2* in brains of naïve mice. Spatial mapping shows that expression of *Tgm1* is restricted to the BO region of the tumor. **(D)** Schematic representation of extracellular matrix alterations in GBM undergoing from a soft to stiff tissue could be regulated by upregulation of ECM proteins such as Tenascin C, Fibronectin, Integrin along with upregulation of a crosslinking protein such Tgm2; *n* = 2 per condition (spatial transcriptomics), *n* = 4 per condition (immunofluorescence).

Composition of extracellular matrix (ECM) in GBM changes during tumor progression due to increased expression of ECM proteins such as laminin and fibronectin, which facilitates diffusive tumor growth [43]. TAAs have been implicated in ECM remodeling and stiffening the matrix [43]. We investigated whether mechanisms that support diffusive tumor growth show an association with the astrocyte heterogeneity. Using Visium ST we mapped cells expressing ECM genes such as *Tenascin C, Laminin b1, Fibronectin 1, Integrin alpha 1,* and found them highly expressed and localized within and around the tumor (Fig 6A). Using IF staining, we visualized increased expression of Fibronectin and Laminin at the TC (Fig 6B) compared to brains of naïve mice. We queried if there are spatial changes in the expression of ECM related genes that could play a role in stabilization of overexpressed ECM proteins leading to stiffening of the matrix. The tissue transglutaminase or transglutaminase 2 (*Tgm2*) is a Ca^2+^-dependent enzyme that cross-links ECM proteins. Reactive astrocytes produce ECM proteins that become a part of the glial scar and TGM2 overexpressed in those cells may contribute to the ECM protein deposition and aggregation [44,45]. We found a robust overexpression of *Tgm2* at the TC and TP in GL261 gliomas, while expression of *Tgm1 (*transglutaminase 1) was confined to the BO (Fig 6C) where it is co-localized with pro-inflammatory markers such as *Lcn2*, *Sipr3* (described in the Fig 1). Similar increased *Tgm2* expression and distribution was detected in two other glioma models, particularly in NRas gliomas (S2 Fig). Therefore, the ECM transition from a soft to stiff tissue in GBM is likely driven by upregulation of ECM proteins such as Fibronectin, Tenascin C, and increased expression of Tgm2 may contribute to their crosslinking and stabilization (Fig 6D).

**Fig 7:**
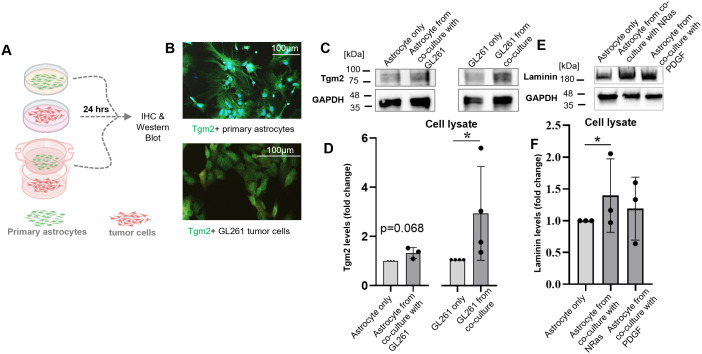
TAAs up-regulate Tgm2 in glioma cells. **(A)** Primary cultures of murine astrocytes and GL261 glioma cells were grown separately or together for 24 h followed by immunohistochemistry and western blot analysis, *n* = 4. **(B)** Representative IF images of astrocytes and GL261 cells showing Tgm2 staining. **(C)** Western blot analysis for Tgm2 from cell lysates of astrocytes, GL261 cells and from those cells growing in co-culture. **(D)** Quantification of Tgm2 protein levels by densitometry of blots demonstrates increased Tgm2 levels in GL261 cells induced by co-culture with astrocytes. Tgm2 levels were analyzed by western blot and densitometry of immunoblots determined from 4 experiments is represented as mean± SD. **(E)** Co-culture of astrocytes with NRas of PDGF glioma cells increases laminin levels in astrocytes. **(F)** Densitometric analysis of immunoblots from three experiments. Data were normalized to the levels of GAPDH in the same sample; control is set as 1; *P* values were calculated using GraphPad on logarithmic values and considered significant when *P < 0.05 (one-way paired t *t*est). The underlying data is available at S2 Data.

We examined the impact of co-culture of primary murine astrocytes with GL261 glioma cells on Tgm2 and Laminin expression. Tgm2 levels were determined by IF and western blot analysis (Fig 7A). Tgm2 was detected by IF staining both in primary astrocyte cultures and GL261 glioma cells (Fig 7B), and Tgm2 protein levels were similar in both cell types. Interestingly, co-culture with astrocytes increased the Tgm2 levels in GL261 cells (Fig 7C and 7D). It shows that activated TAAs by inducing Tgm2 in GL261 cells may regulate ECM reorganization.

Laminin expression was upregulated in primary astrocytes co-cultured with NRAS and PDGF glioma cells as assessed by western blot. In both cases, we detect a robust increase in laminin levels in astrocytes, induced by co-culture glioma cells when compared to controls (Fig 7E and 7F). Spatial transcriptomics analysis and IF analysis show that *Laminin* is overexpressed in GL261 gliomas compared to naïve controls (shown in Fig 6A and 6B). Altogether, the results show that co-culture with astrocytes leads to upregulation of genes coding for ECM proteins such as Fibronectin, Laminin, Tenascin C, and increased expression of Tgm2 in glioma cells may contribute to their crosslinking and stabilization.

### Blockade of tumor-induced reprograming of microglia reduces astrogliosis in TME and restores GLT1 expression in TAAs

**Fig 8:**
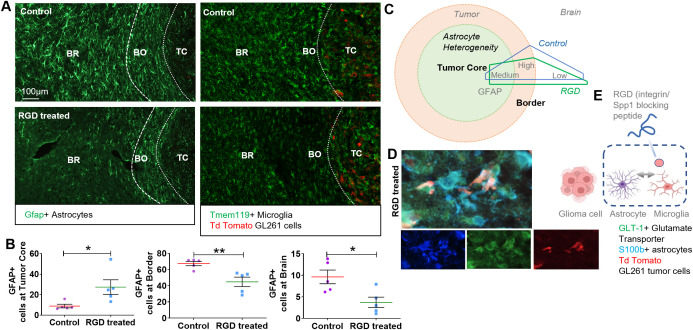
Pharmacologic modification of the tumor microenvironment reduces astrogliosis and harmonizes astrocytic GLT1 expression. **(A)** In RGD-treated mice less Gfap+, reactive astrocytes are detected in the astrocytic ring around the TC and more Gfap+, reactive astrocytes are present in the TC compared to controls. **(B)** Quantification of percentage of area occupied by Gfap+, reactive astrocytes/DAPI at TC, BO, and BR regions. Statistical significance was calculated with one-way paired *t* test, *p < 0.05; **p < 0.01, *n* = 5 per group/condition. The underlying data is available at S3 Data. **(C)** Schematic representation of astrocytic heterogeneity represented by Gfap+, reactive astrocytes at TC, BO, and BR regions and modulation of their distribution in RGD-treated mice. **(D)** Representative image of GLT-1+ with S100b+ TAAs in brain sections of RGD-treated mice depicts harmonization of GLT-1 expression in S100b+ TAAs; *n* = 4 per group/condition. **(E)** Scheme showing that the blockade of integrin signaling by the RGD peptide impacts microglia functions and disrupts their crosstalk with TAAs, which prevents misusing of those cells in tumor progression.

The functional implications of TAAs and formed glial scar in GBM progression are not fully understood. To dissect molecular underpinning of the events, we manipulated the TME using the designer 7aaRGD peptide (shortly RGD) that interferes with glioma-microglia communication and blocks microglia activation via integrin signaling [46–49]. The intratumorally delivered peptide did not affect glioma growth, but blocked the tumor supportive microglia activation and improved immunotherapy with anti-PD1 antibody [49]. This manipulation allowed to study whether a blockade of microglia reprograming, but not tumor growth itself, affects distribution of TAAs. For this purpose, sections from mice receiving intratumoral infusions of the RGD peptide or scrambled control for 21 days were subjected to Gfap staining. In brains of RGD-treated mice, we found a strong reduction of astrogliosis and thickness of the glial scar. Gfap+ astrocytes are significantly more abundant within the TC and reactive Gfap+ astrocytes were reduced at BO and BR areas in the RGD-treated mice compared to controls, which is consistent with the more subdue environment (Fig 8A and 8B). The density of TMEM 119+ microglia population did not change in the tumor core, tumor border, or the adjacent brain region to tumor in the RGD-treated mice compared to controls (Fig 8A). This IF result is consistent with the reported unchanged myeloid compartment as detected by flow cytometry immunophenotyping [49]. However, the substantial changes can be seen in the thickness of glial scar in the RGD treated mice compared to control, indicating microglia targeting with RGD disrupts microglia-astrocyte crosstalk in RGD treated mice and as such this diminishing of glial scar can be an important hallmark to assess TME sensitivity. The schematic model (Fig 8C) we propose shows there is the spatial heterogeneity of Gfap expression in TAAs in various GBM regions. The change in a density of the astrocyte ring suggests its adjustment to the growing tumor, as it is thicker and denser with tumor progression. Markers of TAAs and morphological structure of the glial scar could be important biomarkers of GBM aggressiveness. Blockade of the pro-tumor microglia phenotype reduced activation of TAAs and restored GLT-1 expression in astrocytes residing in the TC in RGD-treated mice likely limiting neuron death (Fig 8D). S100B positive astrocytes are present at the TC, but not GFAP positive ones. The GLT-1 expression do not colocalize at TC with an astrocyte marker S100B. A lack of GLT-1 immune co-localization with S100B at the TC suggests that there are some astrocytes in that location but they do not express GLT-1 which hints for glutamate dysregulation. In RGD-treated mice, we see co-localization of GLT-1 with S100B astrocytes at tumor core, likely reflecting restoration of glutamate homeostasis. Thus, the RGD peptide by affecting tumor-microglia communication restores to some extent homeostatic functions of astrocytes which may result in less tumor supporting TME (Fig 8E). The underlying data is available at S3 Data.

## Discussion

The identification of diverse astrocyte roles in health and disease led to the concept that there are different astrocyte subtypes that exert different functions. Recent advances in scRNA-seq and proteomics enabled the more detailed assessment of molecular differences between cells and allowed to demonstrate that astrocytes do undergo different morphological and molecular transformations related to age, spatial location and disease context. Dissecting complex cross-talks of TAAs with other cells in various areas of GBM and the role of a specific TAA subtype were hampered by a lack of distinctive markers beyond GFAP. The data presented here provide the evidence that distinct populations of TAAs play a critical role in shaping TME of GBM and governing tumor progression. We combined 10× Genomics Visium spatial transcriptomics and multiple marker immunostaining to dissect functional phenotypes of TAAs and comprehend the significance of their localization, as well as to understand a role of specific astrocyte subtypes in the local milieu in GBM. Using specific gene marker profiles (in addition to a well-known marker *Gfap*) we found five distinct transcriptional subtypes of astrocytes based on markers expressed in diverse tumor regions, their distance from the tumor core and unique morphology. These transcriptomic profiles have implications for distinct functions of TAAs in tumor progression. We detected astrocytes expressing classical marker genes (e.g., *Aldh1l1*, *Gfap*, *Aqp4,* and *Slc1a2*) in control brains and their abundance in tumor-bearing hemispheres where they formed a glial scar around the tumor core. These profiles were detected in three murine glioma models, although they were more pronounced in GL261 and NRas gliomas. There was a striking difference between a bipolar shape of Gfap+ TAAs along the tumor core and star-like morphology of astrocytes that were located farther for the tumor core. We demonstrate the maintenance of regional heterogeneity by TAAs in GBM, which adds an additional layer to the known astrocyte heterogeneity described in brain development, aging and inflammation [16,34,50,51]. Re-analysis of the spatially resolved human GBM public dataset showed a similarity in expression pattern of TAAs. *GFAP*, *AQP4, PDPN, LCN2* TAAs form a glial scar around the tumor core, exhibiting region-specific morphologies and gene expression profiles similar to those identified in murine gliomas.

In search for functionalities of TAAs, we found “reactive” TAAs (positive for Gfap) but also *Aldh1l1* expressing TAAs that potentially contribute to glial scar formation, glutamate dysfunction, neurotoxic influence and ECM reorganization via regulation of Tgm2. Aldh1l1 is one of the most reliable and important markers for astrocytes, though its expression did not change in reactive insults [16,34]. A recent study of spatial organization of human GBMs defined the reactive astrocyte cluster characterized by classical astrocytic markers (*AGT* and *GJA1*) and genes coding for metallothionein [27]. Injury-induced glial scar serves as a physical and chemical barrier to axonal regeneration as its major constituents such as Tenascin, N-sulphated heparan sulphate proteoglycans, chondroitin sulfate proteoglycans and keratan sulphate proteoglycans are inhibitory for neurite outgrowth [15,17]. The glial scar in vivo may differ in distribution and type resulting in different biological effects. We found Aldh1l1 expressing, A1 neurotoxic astrocytes to be determinants for regional stratification at the TC of GBM. The spatial data strongly suggests a proximity of microglia in and around the tumor, and its capacity to produce pro-inflammatory cytokines can regulate the behavior of astrocytes. However, we cannot over-rule the possibility of contribution of these cytokines from other sources than microglia, and therefore the results must be interpreted cautiously. Especially, contribution of other myeloid cells and immune components such as dendritic cells, macrophages, monocytes were unexplored due to primary focus of microglia and a shortage of discriminating antibodies for IHC.

We observed changes in expression of genes coding for several ECM proteins (Fibronectin, Laminin B1) in the TC along with the increased expression of *Tgm2*. Upregulated levels of those proteins in the TC confirmed by IF demonstrate a robust remodeling of the tumor core. The localized expression of *Tgm2* in the TC suggests its role in the reinforcing the ECM re-organization. Tgm2 crosslinks various ECM proteins, including fibronectin, fibrinogen/fibrin, von Willebrand factor, vitronectin, dermatan sulfate proteoglycans, collagen V, osteonectin, laminin and osteopontin. Astrocytic Tgm2 has been shown to facilitate cell migration and proliferation, reducing their ability to protect neurons after brain injury. Moreover, Tgm2 supports glioma stemness and radioresistance [44]. We found that glioma cells increased levels of Tgm2 in the presence of TAAs. As Tgm2 regulates ECM stiffening by altering the composition of ECM proteins, these properties make the enzyme an important target for GBM therapy.

Experimental gliomas (GL261 and genetically engineered models) develop rapidly in syngeneic mouse brains, thus many parameters such as invasiveness, stemness, proliferation, migration, therapy resistance and signaling pathways can differ between murine and human glioma cells. GL261 gliomas may not fully recapitulate the stiffness and ECM complexity seen in human gliomas, but the presented ST studies show significant upregulation of genes coding for Fibronectin, Laminin and Tenascin-C in murine and in human gliomas. In NRas and PDGF glioma models, we also identified similar profiles of upregulation of genes coding for Laminin or Tgm2 as seen in GL261 gliomas. Thus, these mouse glioma models could be useful for studying ECM reorganization and investigating related mechano-transduction pathways.

Using a recently established model for manipulation of glioma TME, in which a synthetic peptide RGD blocks glioma-microglia communication and restores homeostatic microglia in TME, we explored microglia-TAAs interactions. We show that TAAs are modulated as a consequence of the RGD treatment and changed their morphology at the glial scar, restoring the less reactive phenotype. The glial scar forms a physical barrier for cell–cell communication, cell infiltration, or even for drug penetration [19,52,53]. We found that in RGD-treated tumors the glial scar is less dense with more astrocytes within the TC. Those TAAs express GLT-1 that suggests a normalizing effect on TME.

Spot-based spatial transcriptomics employed in this study has its limitations such as low resolution, making resolving individual cell states, and cell types challenging. Thus, we cannot completely rule out the chances of overlap of astrocytic markers between TAAs and GBM cells as a limitation in this study. Though with IF we could see distinct expression of Aldh1l1 or S100b on astrocytes (recognized by specific morphology) around fluorescently labeled GBM cells. S100b staining also co-localized with Gfap expression in IF analysis. However, a recent study showed that cells with similar expression group and tend to be surrounded by other cells in the same state forming local environments, highly enriched with cells in an individual state. While the 10X Visium technology does not provide single-cell resolution, as a diameter of a spot is around 55 μm, they found a spot contains a mixture of 1–35 cells, with a median of 8 cells in GBM based on image analysis [27]. Despite this limitation, the transcriptomic patterns detected in this study provide meaningful assumptions and most of the findings were confirmed by multiple staining for astrocytic markers. Herein, by combining spatial transcriptomics and immunostainings, we resolved astrocytic heterogeneity in three glioma models and human GBMs, and demonstrated astrocyte regulation within TME and its impact on glial scar formation and tumor expansion. The experiments with the RGD peptide modifying TME allowed to dissect microglia and TAAs interactions and comprehend more precisely their impact on other cells.

All data supporting the findings of this study are available within the manuscript or the supplementary information. The raw spatial transcriptomics data are available in the GEO under accession number GSE269545.

## Supporting information

S1 Fig Spatial transcriptomic analysis of other important marker genes associated with diverse functions of astrocytes. ST data for selected marker genes are visualized in tumor bearing brains (upper panel) and controls (lower panel); *n* = 2 mice/condition.
(TIF)

S2 Fig Spatial heterogeneity of astrocyte markers in murine NRas and PDGF gliomas. ST data for selected marker genes are visualized; each replicate is presented individually; *n* = 2 mice/condition.
(TIF)

S3 Fig Spatial co-occurrence of cell types across tumor regions and their differential comparison. Heatmaps display the pairwise co-occurrence scores of deconvoluted cell types (astrocytes, microglia, tumor cells) across three spatial regions of the tumor: core, periphery, and border (top panel). Co-occurrence values range from −1 (mutual spatial avoidance, blue) to +1 (strong spatial co-localization, red), with 0 indicating no spatial correlation (white). Each square reflects the degree of spatial overlap between a pair of cell types within the specified region. Bottom panel (B–D) shows the differential co-occurrence between tumor regions. Panel **B** depicts the difference in co-occurrence between the core and periphery, **C** between the core and border, and **D** between the periphery and border. Color intensity represents the magnitude and direction of change in co-occurrence (orange for increased, blue for decreased co-localization in the second region compared to the first). Notably, tumor cells co-occur more strongly with astrocytes in the periphery than in the core, and microglia–tumor interactions show reduced co-occurrence in the border compared to the core.
(TIF)

S1 Data Raw data on the correlation of cells expressing *C3* with astrocytic markers *Gfap, Aldh1l1, S100b* at different tumor regions; Fig 3F. (XLSX)

S2 Data Raw data from densitometric analysis of immunoblots showing Tgm2 and GAPDH levels from 3 experiments in the Fig 7C and 7F. (PDF)

S3 Data Raw data from quantification of percentage of area occupied by Gfap+, reactive astrocytes/DAPI in different tumor regions: at TC, BO, and BR in the Fig 8. (XLSX)

S1 Raw Images Original blot and gel images contained in Fig 7C and 7E. (PDF)

## Abbreviations

BBB: blood–brain barrier
BO: border
DMEM: Dulbecco’s modified Eagle’s medium
ECM: extracellular matrix
GBM: glioblastoma
GSEA: gene-set enrichment analysis
H&E: hematoxylin and eosin
IF: immunofluorescence
IHC: immunohistochemistry
PBS: phosphate-buffered saline
PCA: principal component analysis
scRNA-seq: single-cell RNA sequencing
TAAs: tumor-associated astrocytes
TC: tumor core
tdT: tandem Tomato
TME: tumor microenvironment
TNF: tumor necrosis factor
TP: tumor periphery
UMAP: uniform manifold approximation and projection
