# Supplementary figures and images for: Identification and characterization of tumor-associated astrocyte subpopulations and their interactions with the tumor microenvironment in experimental glioblastomas
Source: PLoS Biol. 2025 Oct 13;23(10):e3002893. doi: 10.1371/journal.pbio.3002893 (PMC12539703; doi:10.1371/journal.pbio.3002893)

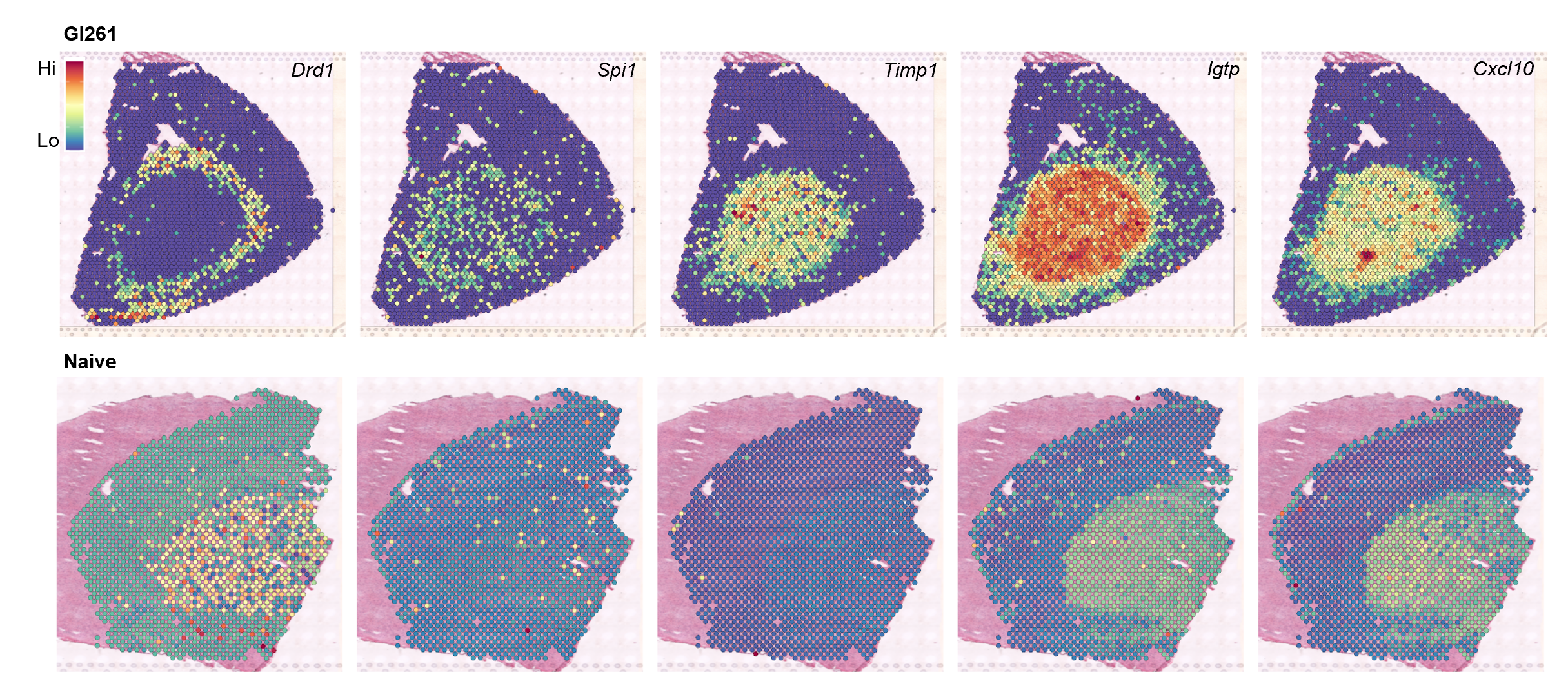

Supplement: S1 Fig — ST data for selected marker genes are visualized in tumor bearing brains (upper panel) and controls (lower panel); n = 2 mice/condition. (TIF) [file pbio.3002893.s001.tif]

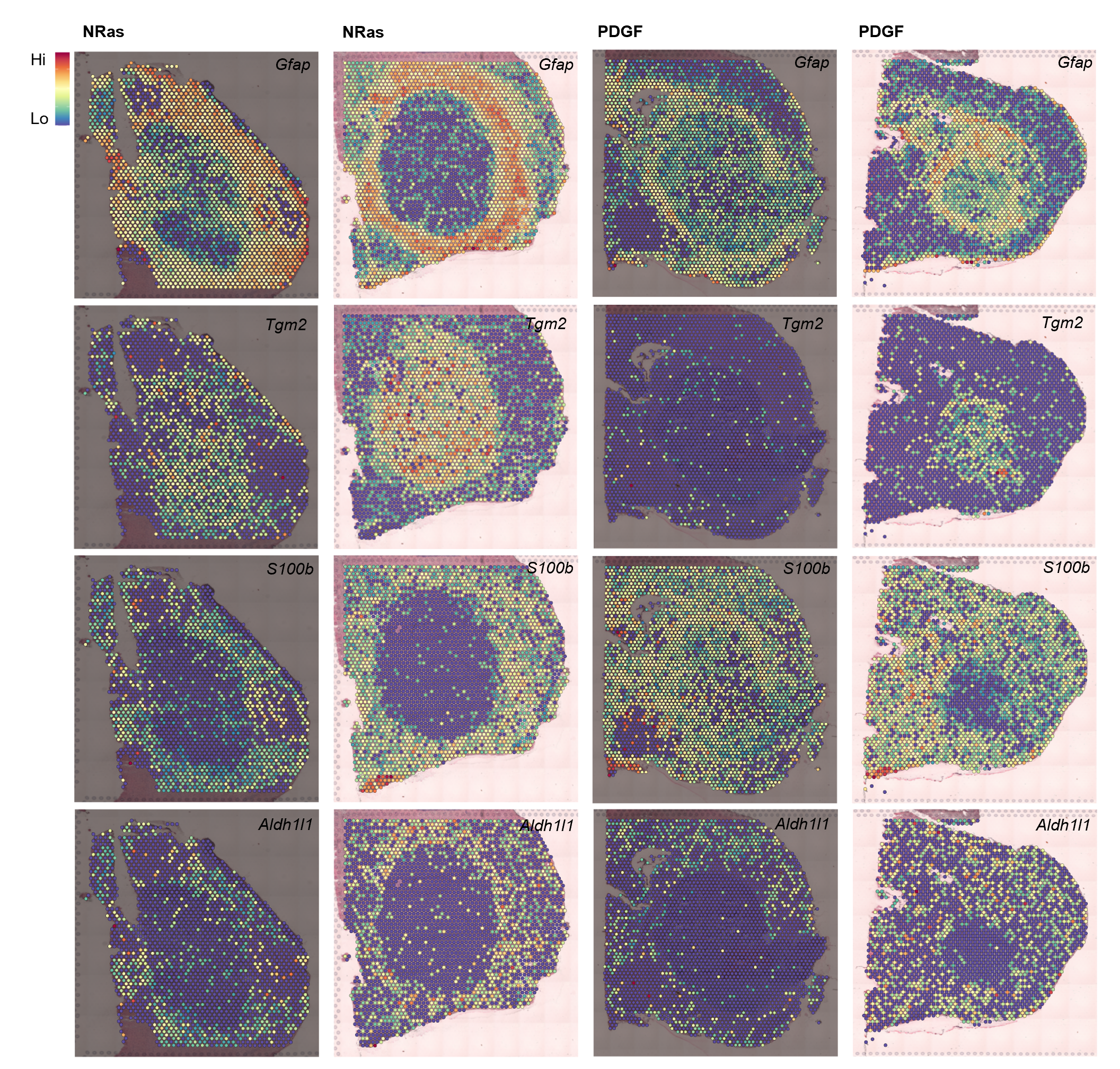

Supplement: S2 Fig — ST data for selected marker genes are visualized; each replicate is presented individually; n = 2 mice/condition. (TIF) [file pbio.3002893.s002.tif]

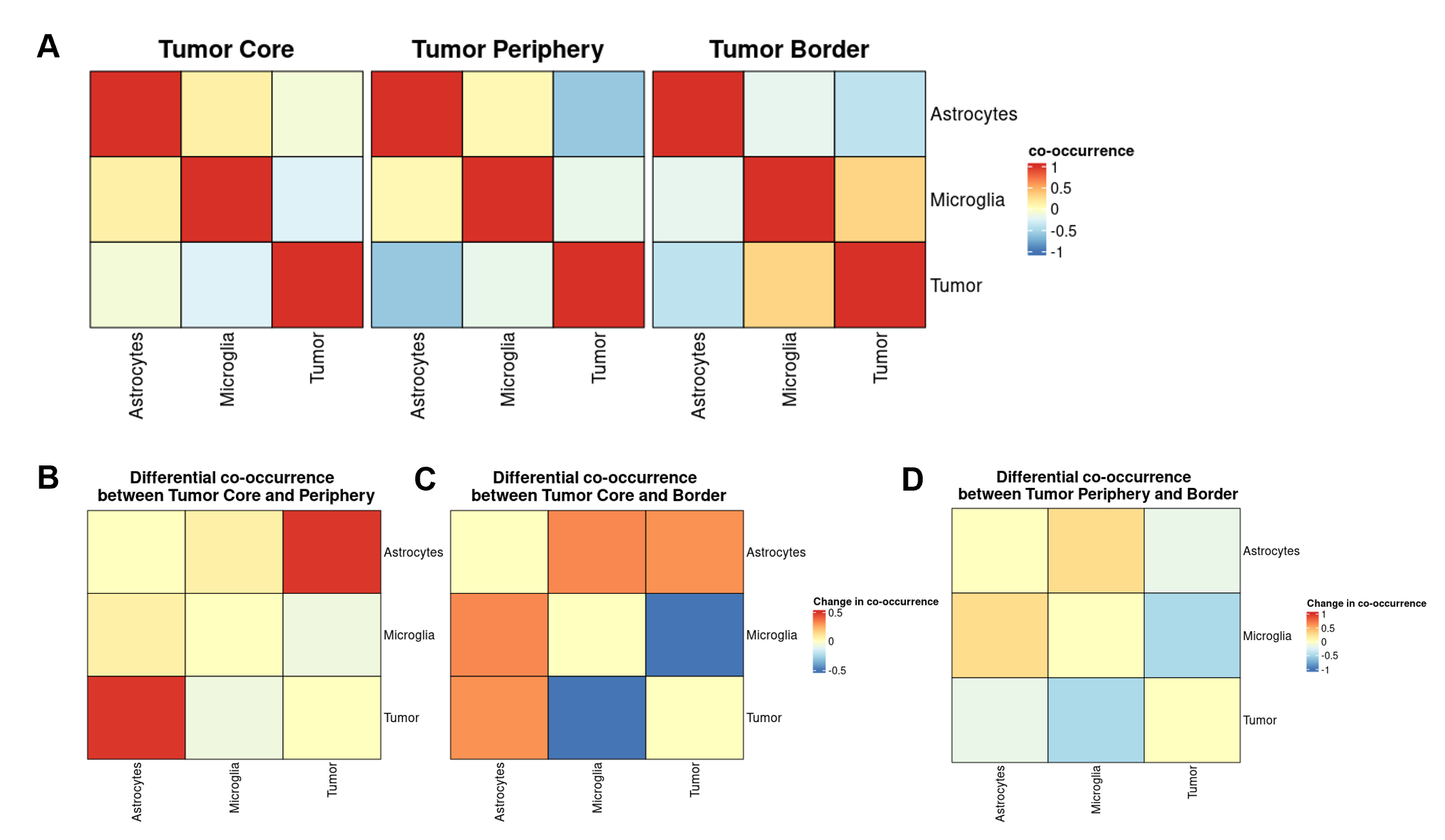

Supplement: S3 Fig — Heatmaps display the pairwise co-occurrence scores of deconvoluted cell types (astrocytes, microglia, tumor cells) across three spatial regions of the tumor: core, periphery, and border (top panel). Co-occurrence values range from −1 (mutual spatial avoidance, blue) to +1 (strong spatial co-localization, red), with 0 indicating no spatial correlation (white). Each square reflects the degree of spatial overlap between a pair of cell types within the specified region. Bottom panel (B–D) shows the differential co-occurrence between tumor regions. Panel B depicts the difference in co-occurrence between the core and periphery, C between the core and border, and D between the periphery and border. Color intensity represents the magnitude and direction of change in co-occurrence (orange for increased, blue for decreased co-localization in the second region compared to the first). Notably, tumor cells co-occur more strongly with astrocytes in the periphery than in the core, and microglia–tumor interactions show reduced co-occurrence in the border compared to the core. (TIF) [file pbio.3002893.s003.tif]
