# Supplementary material for: Identification and characterization of tumor-associated astrocyte subpopulations and their interactions with the tumor microenvironment in experimental glioblastomas
Source: PLoS Biol. 2025 Oct 13;23(10):e3002893. doi: 10.1371/journal.pbio.3002893 (PMC12539703; doi:10.1371/journal.pbio.3002893)
Supplement: S2 Data — (PDF) [file pbio.3002893.s005.pdf]

Figure 7C

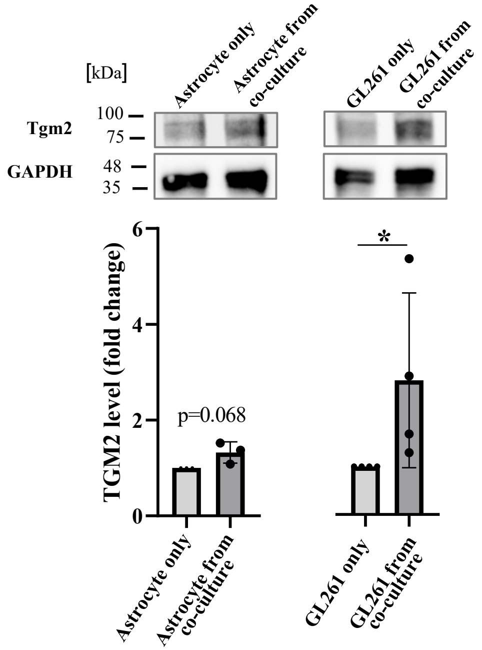
[kDa]
Astrocyte only
Astrocyte from co-culture
GL261 only
GL261 from co-culture
Tgm2
GAPDH
100
75
48
35
TGM2 level (fold change)
6
4
2
0
p=0.068
*
Astrocyte only
Astrocyte from co-culture
GL261 only
GL261 from co-culture

Figure 7C

Analysis if co-culture of astrocytes and GL261 increases the levels of TGM2

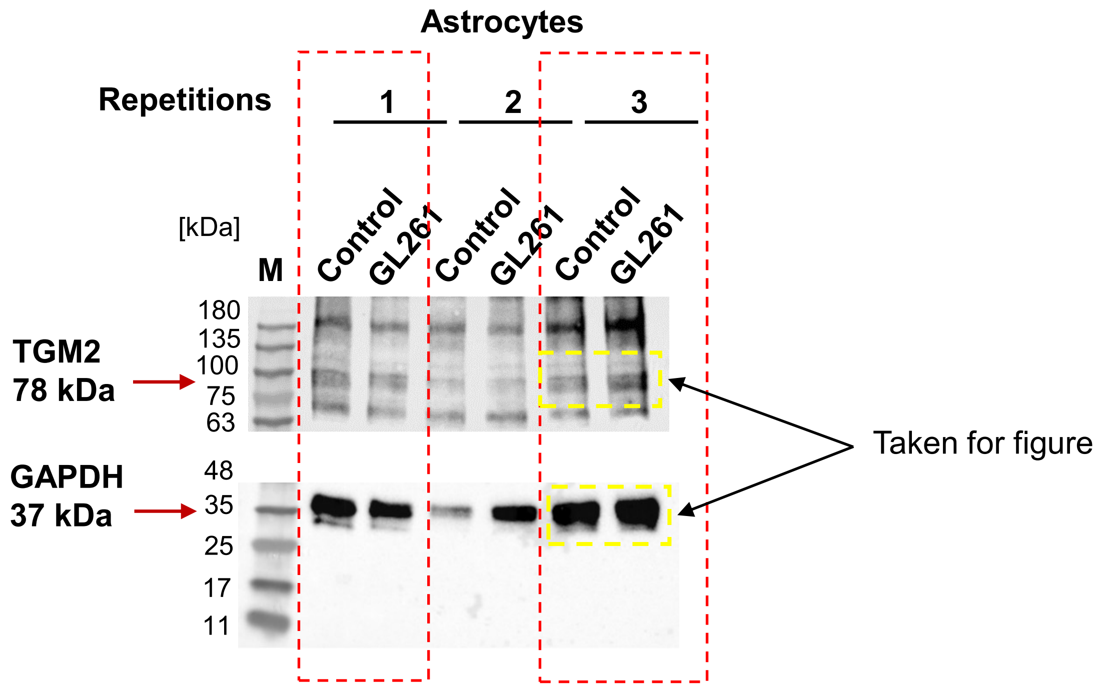

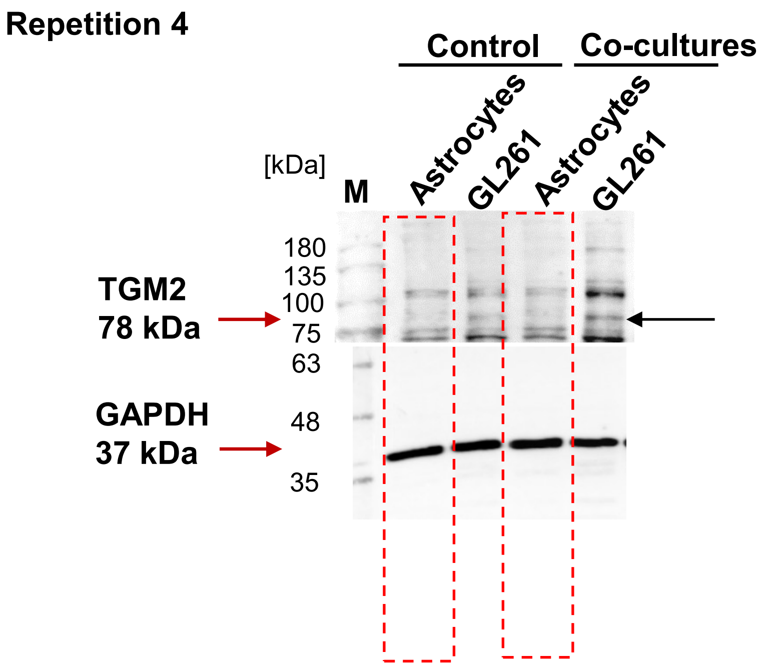

Note: Repetition 2 was rejected because there was not enough of sample and GAPDH was unequal.

Figure 7C

Analysis if co-culture of astrocytes and GL261 increases the levels of TGM2

Repetition	Astrocytes						
	Adj. Vol. Corrected for GAPDH		Log		FC		
	Control	Co-culture	Control	Co-culture	Control	Co-culture	
1 (27.10.23)	800094	1217627	5,903141	6,085514	1	1,52	
2 (02.11.23)	*275349*	*52730*	*5,439884*	*4,722058*	*1*	*0,19*	Excluded
3 (23.11.23)	644023	694855	5,808901	5,841894	1	1,08	
4 (17.11.22)*	641607	880995	5,807269	5,944973	1	1,37	

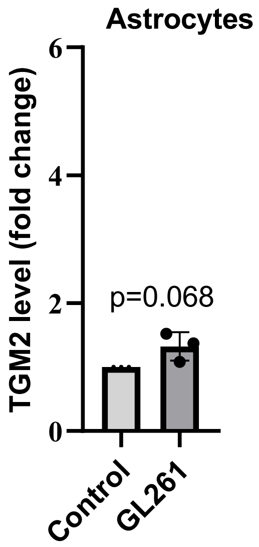

Paired t test<br>Tabular results	
Table Analyzed	Astrocytes* GL261 log
Column B	GL261
vs.	vs.
Column A	Control
**Paired t test**	
P value	0.0586
P value summary	ns
Significantly different (P < 0.05)?	No
One- or two-tailed P value?	One-tailed
t, df	t=2.659, df=2
Number of pairs	3
**How big is the difference?**	
Mean of differences (B - A)	0.1177
SD of differences	0.07667
SEM of differences	0.04427
95% confidence interval	-0.07278 to 0.3082
R squared (partial eta squared)	0.7794
**How effective was the pairing?**	
Correlation coefficient (r)	0.9005
P value (one tailed)	0.1432
P value summary	ns
Was the pairing significantly effective	No

Figure 7C

Analysis if co-culture of astrocytes and GL261 increases the levels of TGM2

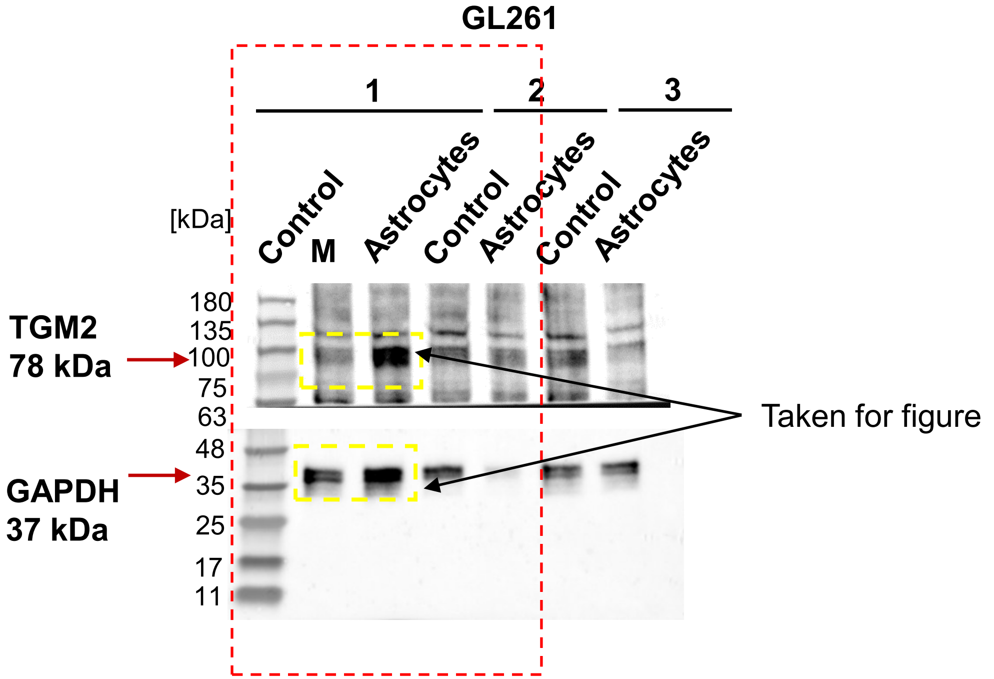

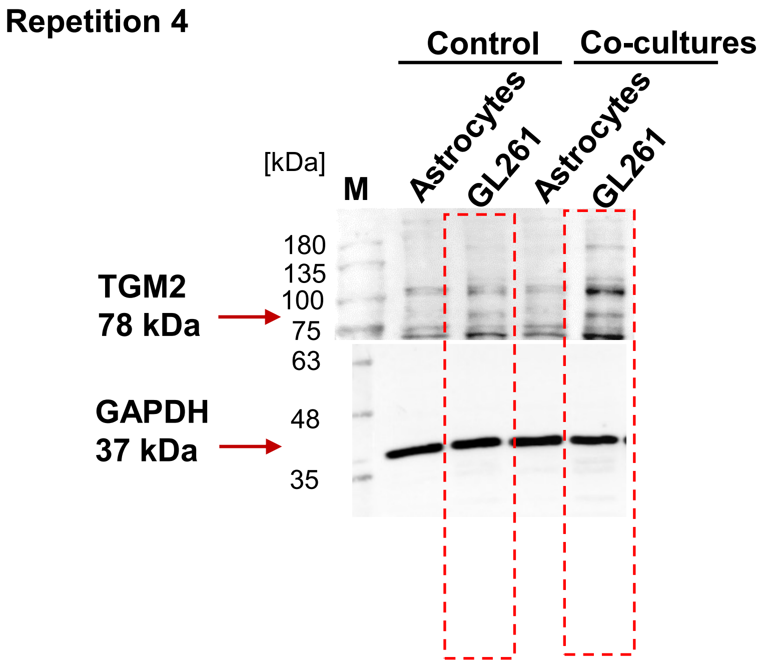

Note: Repetition 3 was rejected because there was not enough sample.

Figure 7C

Analysis if co-culture of astrocytes and GL261 increases the levels of TGM2

Repetition 5

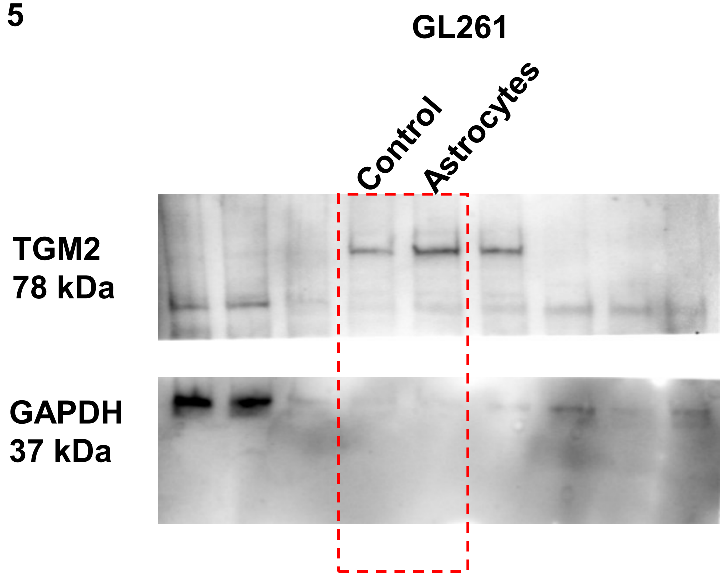

Figure 7C

Analysis if co-culture of astrocytes and GL261 increases the levels of TGM2

Repetition	GL261						
	Adj. Vol. Corrected for GAPDH		Log		FC		
	Control	Co-culture	Control	Co-culture	Control	Co-culture	
1 (27.10.23)	1518897	1960594	6,181528	6,292388	1	1,29	
2 (02.11.23)	1431031	7639766	6,155649	6,88308	1	5,34	
3 (23.11.23)	*1690852*	*1251538*	*6,228106*	*6,097444*	*1*	*0,74*	Excluded
4 (17.11.22)*	965870	2788014	5,984919	6,445295	1	2,89	
5 (09.08.23)*	187138	315269	5,272162	5,498681	1	1,68	

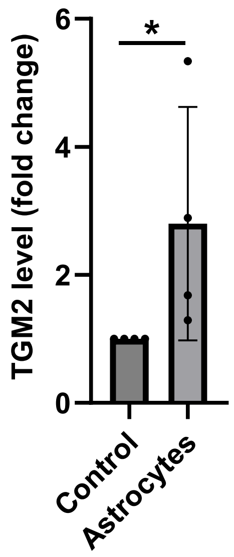

Paired t test<br>Tabular results	
Table Analyzed	GL261* Astrocytes log
Column B	Astrocytes
vs.	vs.
Column A	Control
**Paired t test**	
P value	0.0340
P value summary	*
Significantly different ($P < 0.05$)?	Yes
One- or two-tailed P value?	One-tailed
t, df	t=2.796, df=3
Number of pairs	4
**How big is the difference?**	
Mean of differences (B - A)	0.3813
SD of differences	0.2727
SEM of differences	0.1364
95% confidence interval	-0.05269 to 0.8153
R squared (partial eta squared)	0.7227
**How effective was the pairing?**	
Correlation coefficient (r)	0.8955
P value (one tailed)	0.0523
P value summary	ns
Was the pairing significantly effective?	No

Figure 7E

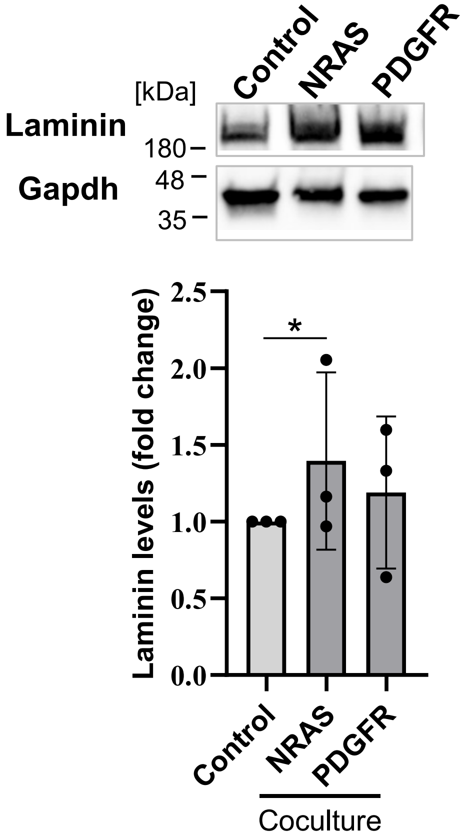
Control
NRAS
PDGFR
[kDa]
Laminin
180
Gapdh
48
35
2.5
2.0
1.5
1.0
0.5
0.0
*
Laminin levels (fold change)
Control
NRAS
PDGFR
Coculture

Figure 7E

Analysis if co-culture of astrocytes and GL261 increases the levels of laminin

Note: The same samples were run twice on Western blot and a median was made of the results from these two technical repetitions.

Technical 1 repetition

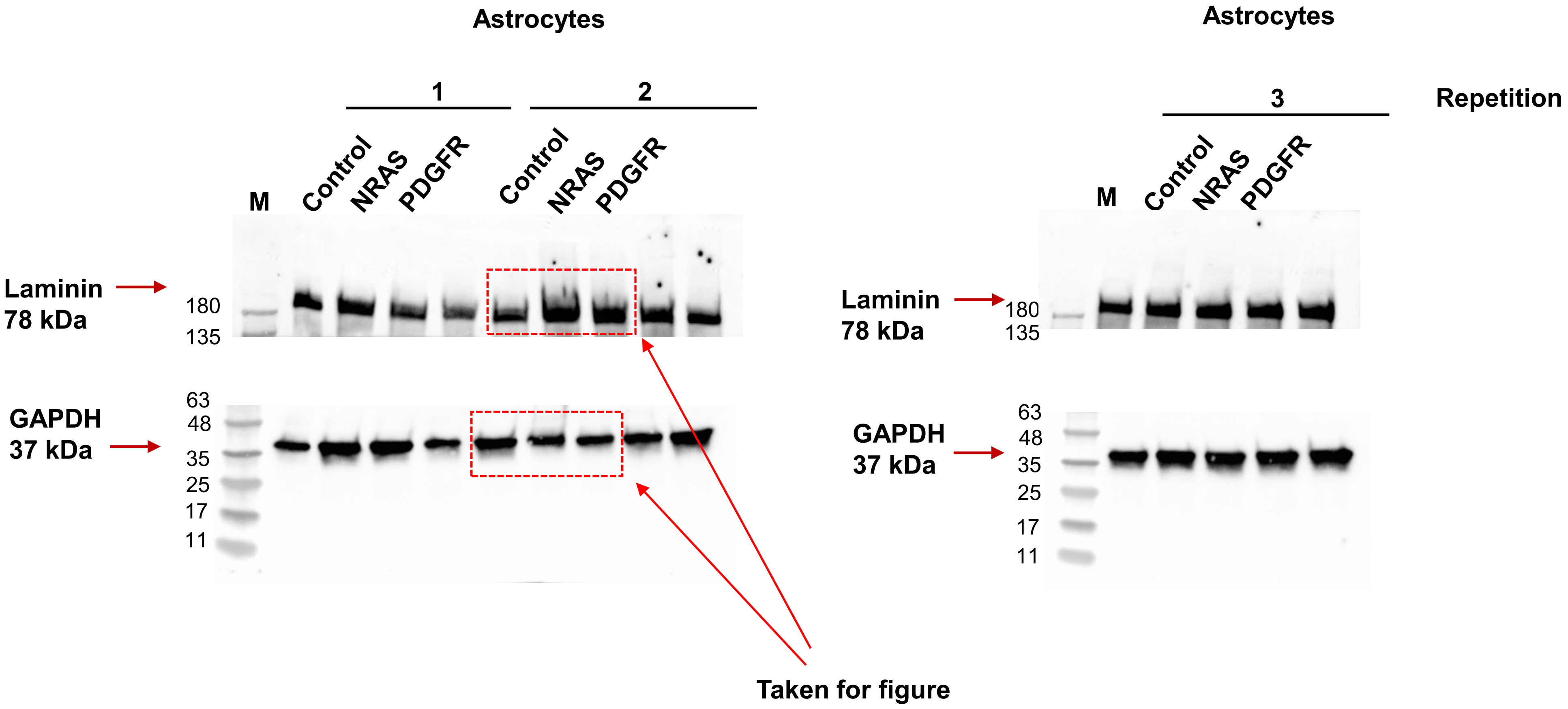

Figure 7E

Analysis if co-culture of astrocytes and GL261 increases the levels of laminin

Note: The same samples were run twice on Western blot and a median was made of the results from these two technical repetitions.

Technical 2 repetition

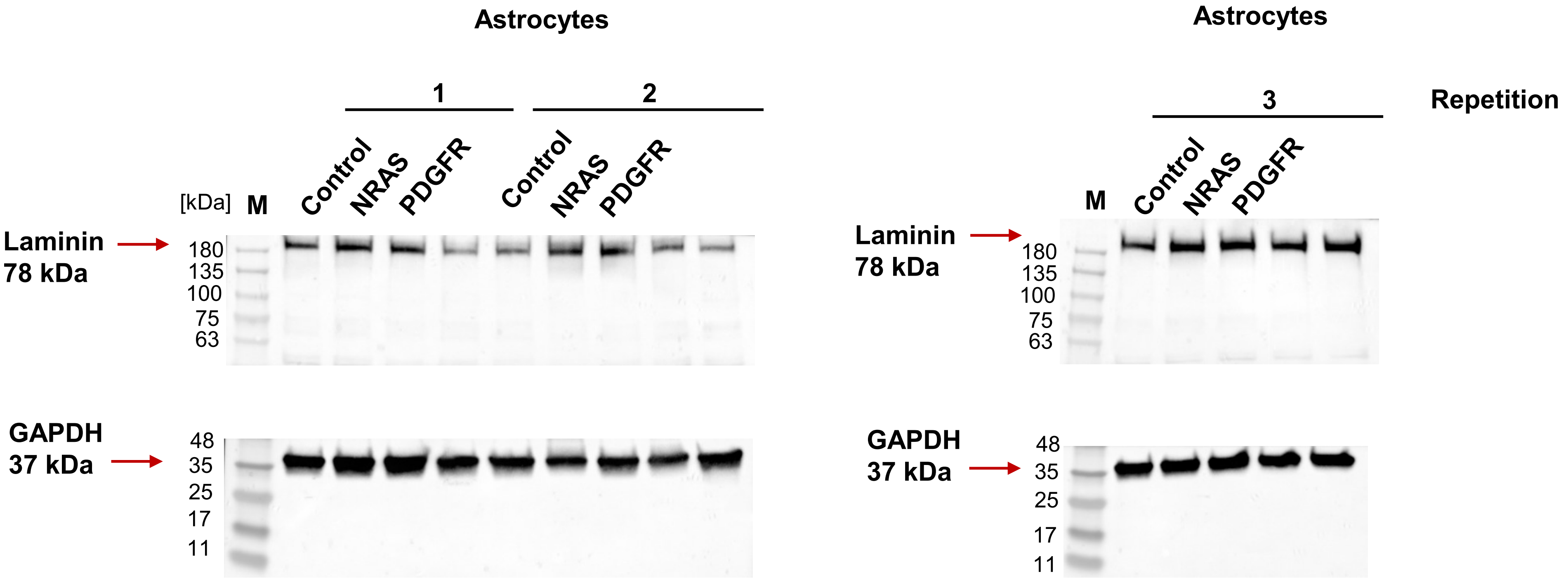

Figure 7E

Analysis if co-culture of astrocytes and GL261 increases the levels of laminin

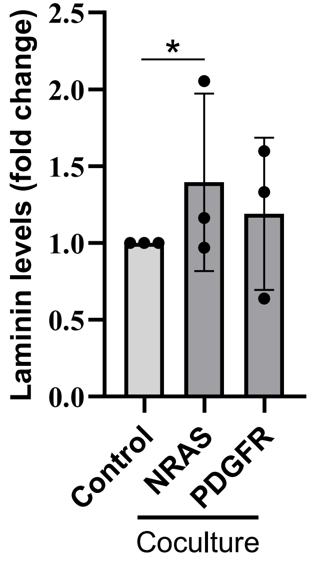

Median									
Repetitions	Adj. Volume corrected for GAPDH			FC			Log		
	A/-	A*/NRAS	A*/PDGFR	A/-	A*/NRAS	A*/PDGFR	A/-	A*/NRAS	A*/PDGFR
1	13164591	21605540	19474948,1	1	1,0	0,8	7,1	7,3	7,2
2	17834971	18380221	15699288,55	1	2,1	1,8	7,2	7,3	7,2
3	17524646	21563219	20234890,95	1	1,3	1,3	7,2	7,3	7,3

RM one-way ANOVA<br>Multiple comparisons					
Number of families	1				
Number of comparisons per family	2				
Alpha	0.05				
**Dunnett's multiple comparisons test**	**Mean Diff.**	**95.00% CI of diff.**	**Below threshold?**	**Summary**	**Adjusted P Value**
Control vs. NRAS	-0.1230	-0.2420 to -0.004012	Yes	*	0.0470
Control vs. PDGFR	-0.06909	-0.1726 to 0.03442	No	ns	0.1052
**Test details**	**Mean 1**	**Mean 2**	**Mean Diff.**	**SE of diff.**	**n1**
Control vs. NRAS	7.157	7.280	-0.1230	0.02196	3
Control vs. PDGFR	7.157	7.226	-0.06909	0.01910	3
