# Supplementary material for: Identification and characterization of tumor-associated astrocyte subpopulations and their interactions with the tumor microenvironment in experimental glioblastomas
Source: PLoS Biol. 2025 Oct 13;23(10):e3002893. doi: 10.1371/journal.pbio.3002893 (PMC12539703; doi:10.1371/journal.pbio.3002893)

Figure 7C

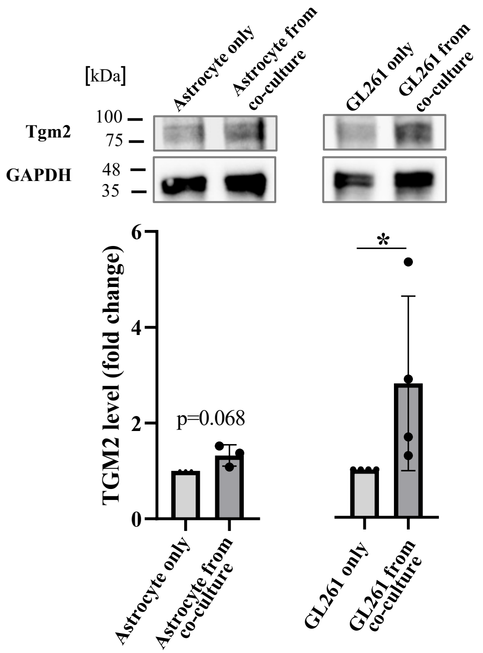

Uncropped Western blot detected with Chemidoc

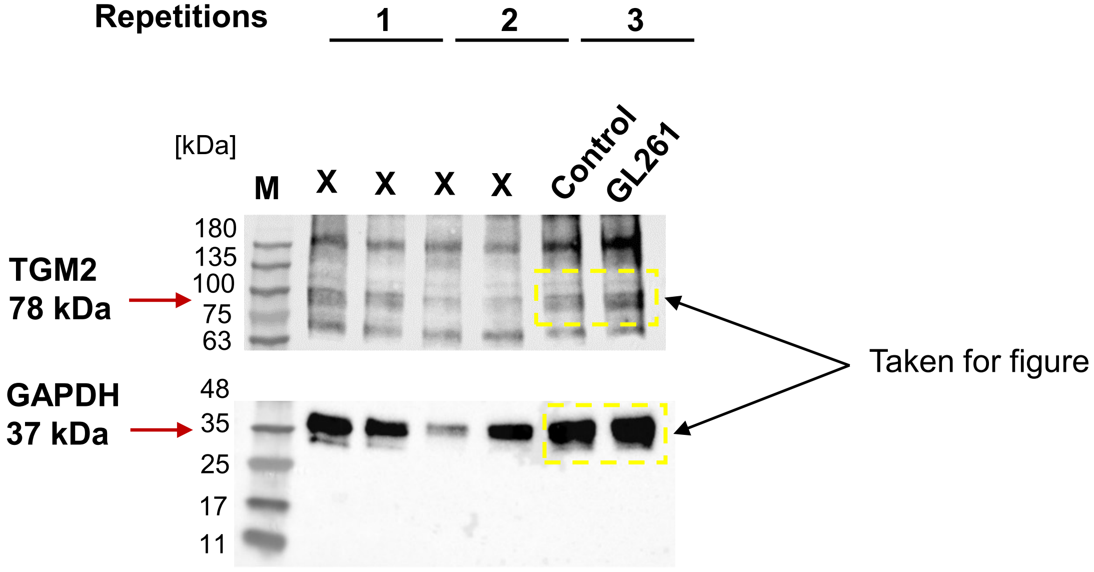

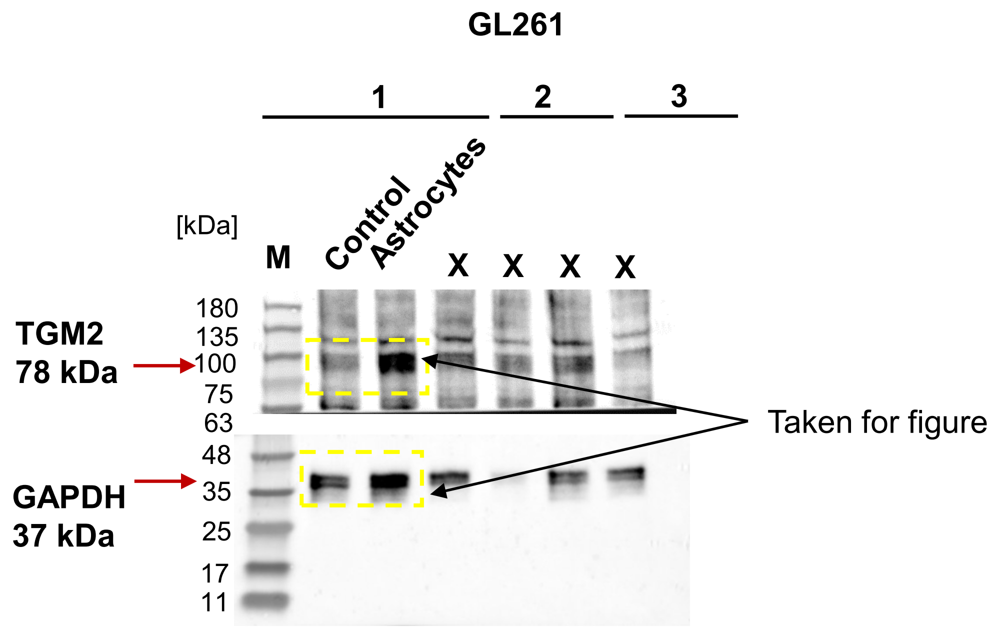

Figure 7E

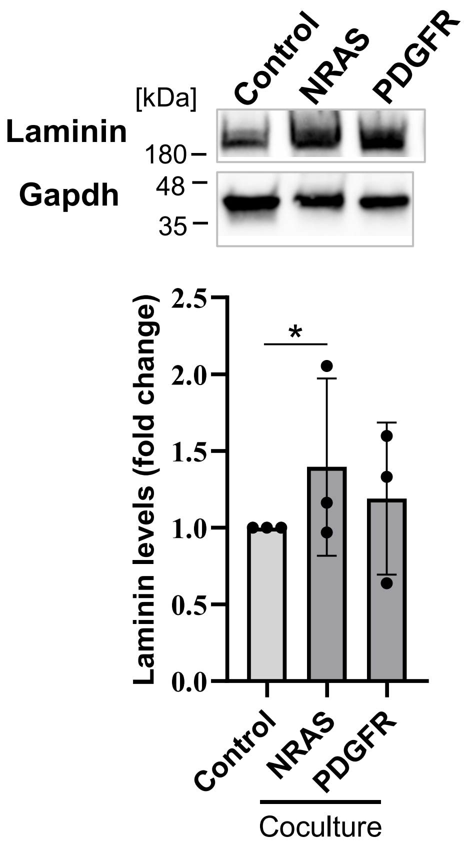

Uncropped Western blot detected with Chemidoc

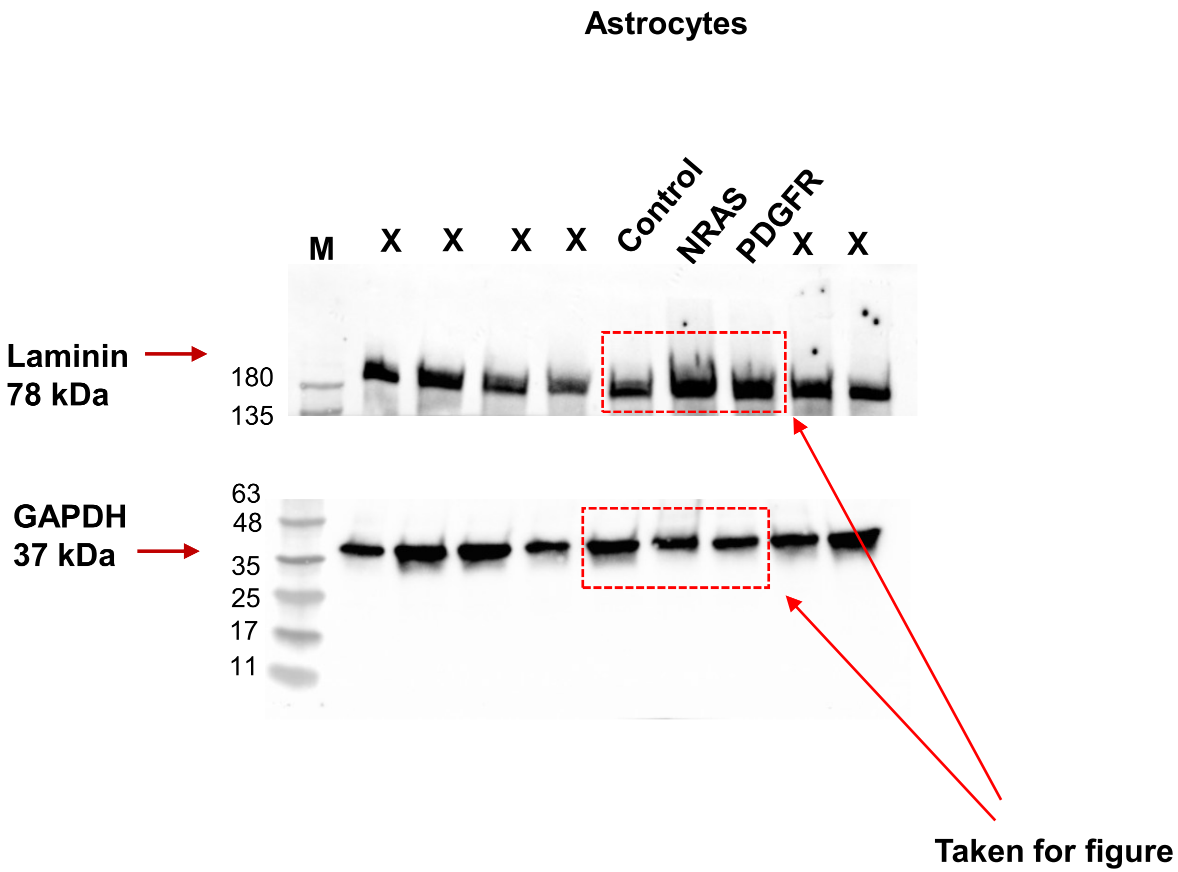

Supplement: S1 Raw Images — (PDF) [file pbio.3002893.s007.pdf]
